# Unifying the Conversation: Membrane Separation Performance in Energy, Water, and Industrial Applications

**DOI:** 10.1021/acsestengg.3c00475

**Published:** 2024-01-26

**Authors:** Sarah M. Dischinger, Daniel J. Miller, David A. Vermaas, Ryan S. Kingsbury

**Affiliations:** † Chemical Sciences Division, Lawrence Berkeley National Laboratory, Berkeley, California 94720, United States; ‡ Department of Chemical Engineering, Delft University of Technology, 2629HZ Delft, The Netherlands; § Energy Storage and Distributed Resources Division, Lawrence Berkeley National Laboratory, Berkeley, California 94720, United States; ∥ Department of Civil and Environmental Engineering and the Andlinger Center for Energy and the Environment, Princeton University, Princeton, New Jersey 08540, United States

**Keywords:** Membranes, Selectivity, Permeability, Chemical potential, Separation mechanism

## Abstract

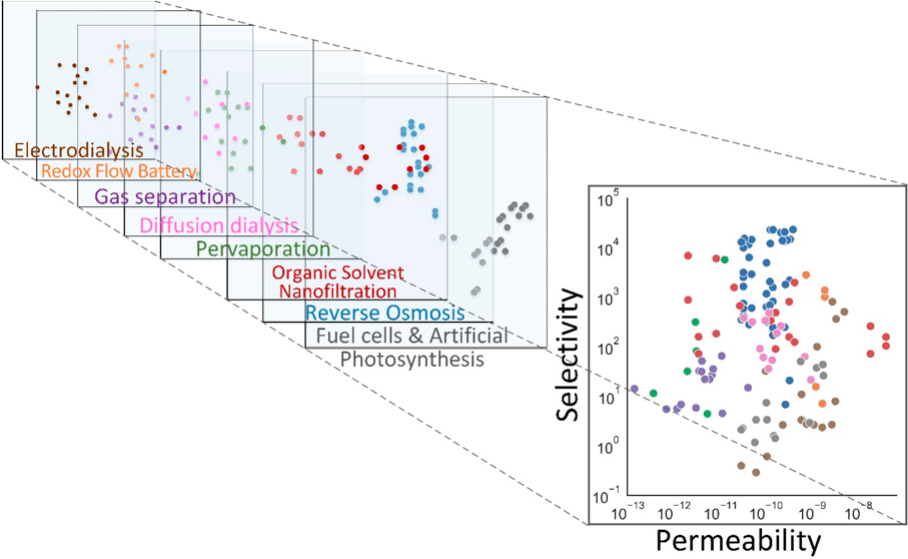

Dense polymer membranes enable a diverse range of separations and clean energy technologies, including gas separation, water treatment, and renewable fuel production or conversion. The transport of small molecular and ionic solutes in the majority of these membranes is described by the same solution-diffusion mechanism, yet a comparison of membrane separation performance across applications is rare. A better understanding of how structure–property relationships and driving forces compare among applications would drive innovation in membrane development by identifying opportunities for cross-disciplinary knowledge transfer. Here, we aim to inspire such cross-pollination by evaluating the selectivity and electrochemical driving forces for 29 separations across nine different applications using a common framework grounded in the physicochemical characteristics of the permeating and rejected solutes. Our analysis shows that highly selective membranes usually exhibit high solute rejection, rather than fast solute permeation, and often exploit contrasts in the size and charge of solutes rather than a nonelectrostatic chemical property, polarizability. We also highlight the power of selective driving forces (e.g., the fact that applied electric potential acts on charged solutes but not on neutral ones) to enable effective separation processes, even when the membrane itself has poor selectivity. We conclude by proposing several research opportunities that are likely to impact multiple areas of membrane science. The high-level perspective of membrane separation across fields presented herein aims to promote cross-pollination and innovation by enabling comparisons of solute transport and driving forces among membrane separation applications.

## 1 Introduction

Polymer membranes enable a diverse array of separations in water purification, wastewater treatment, power generation, energy storage, and chemical manufacturing. Compared to heat-driven separations, which currently account for approximately 10% of world energy consumption, membrane separations can be as much as an order of magnitude more energy efficient^1^ and, hence, can play a major role in the energy transition.

Strong interest in membrane technology is signaled by the more than 86,000 scientific publications and nearly half a million patents related to membranes published in the past decade (based on search results from ISI Web of Science and Google Patents, respectively; see SI for search terms). However, despite vigorous interest, the estimated annual rate of technological improvement in membrane separations is only 10.3% (based on search results from Technology Search Portal, see SI for details), notably less than the average of 19%.^2^ We believe one major factor hindering rapid progress is a siloed research approach in which developments that benefit one application are not often translated to other applications, despite considerable similarity in the materials and physical principles involved. Cross-pollination of ideas across disparate applications is a significant driver of technology improvements^2,3^ that could dramatically accelerate innovation rates in membrane separation technologies. Although there have been a few recent efforts to translate developments across membrane processes, notably using reverse osmosis (RO) membranes in water electrolysis,^4^ redox flow batteries,^5^ and electrodialysis,^6,7^ and comparing properties of ion exchange properties across diverse applications,^8^ such cross-pollination in membrane science remains the exception rather than the rule.

RO,^9−11^ gas separation (GAS),^9,11−13^ organic solvent nanofiltration (OSN),^14^ pervaporation (PV),^9,11^ diffusion dialysis (DD),^9,15^ electrodialysis (ED),^15−17^ fuel cells (FC),^18^ artificial photosynthesis (AP),^19^ and redox flow batteries (RFBs)^15^ all utilize dense, nonporous, amorphous, polymeric membranes through which small molecular and ionic solutes selectively permeate by a common solution-diffusion mechanism. Many of these applications also involve the same solutes. For example, membranes in both PV and FC applications reject methanol (MeOH). Yet, the common figures of merit for membrane performance are highly application-specific (Table 1),^20−25^ impeding comparisons across applications and thereby inhibiting knowledge transfer.^26^ The highly selective membranes used in commercially successful processes such as RO and some gas separations^27^ stand in contrast to the cost-prohibitive, moderately selective membranes^28−33^ used in ED and RFB, suggesting that much could be gained from increased dialogue across disciplines.

**Table 1:** Selected Applications of Dense Polymer Membranes, Organized by a Typical Figure of Merit for Membrane Selectivity^a^

*Application*	*Typical Permeating Solute*	*Typical Rejected Solute*
*Figure of Merit: **% Rejection***		
Reverse Osmosis (RO)	H_2_O	monovalent salt (NaCl) small solutes (B(OH)_3_, As(III))
Diffusion Dialysis (DD)	acid (HCl)	metal salt (Cu, Ni, Zn)
Organic Solvent Nanofiltration (OSN)	small organics (MeOH, EtOH)	molecular products
*Figure of Merit: **Permselectivity***		
Gas (GAS)	CO_2_, O_2_	CH_4_, N_2_
Pervaporation (PV)	H_2_O	monovalent salts (NaCl) small organics (MeOH, EtOH)
Electrodialysis (ED)	counterions (Na^+^, Cl^–^)	co-ions (Cl^–^, Na^+^)
Figure of Merit:		
Fuel Cells (FC)	charge carriers (H^+^, OH^–^)	fuel (MeOH, H_2_)
Artificial Photosynthesis (AP)	charge carriers (H^+^, OH^–^)	CO_2_ reduction products (CH_2_O_2_, MeOH)
Redox Flow Batteries (RFB)	charge carriers (H^+^, OH^–^)	redox species (VO^2+^, Br^–^, Fe^2+^)

a Typical permeating and rejected solutes, which pass through and are blocked by the membrane, respectively, are also shown.

In this perspective, we aim to foster such a cross-disciplinary dialogue by presenting direct comparisons of membrane separation performance across nine applications, comprising 29 individual separations (i.e., pairs of permeating and rejected solutes). We use this data set to perform a high level (order-of-magnitude) comparison of membrane performance across diverse separations and develop a conceptual framework for understanding separation performance based on the applied driving force (e.g., pressure, electricity, etc.) and the size, charge, and other chemical properties of the solutes. We conclude with case studies that illustrate how this conceptual framework applies to several predominant membrane applications and identify opportunities for collaborative membrane development.

## 2 Enabling Comparisons among Disparate Membrane Applications

We compiled membrane separation data from 48 studies comprising 29 distinct separations and 70 distinct membrane types in nine applications (Table 1). Although conventions for reporting data varied widely, the transport of a solute *i* through a dense membrane can always be quantified by its flux *J*_*i*_ (mol·m^–2^·s^1^), which is the net result of (1) the electrochemical potential driving force across the membrane and (2) resistance to transport imposed by the membrane material. These factors can be quantitatively related by^11,34^
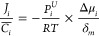
1where  (mol·L^–1^) is the average concentration of the solute in the external fluids (upstream and downstream), *P*_*i*_^*U*^ (m^2^·s^–1^) is the membrane permeability, *R* (8.314 J·mol^–1^·K^–1^) is the ideal gas constant, *T* (K) is the absolute temperature, Δμ_*i*_ (kJ·mol^–1^) is the electrochemical potential, and δ_*m*_ (m) is the membrane thickness (the active layer only, in the case of asymmetric and multilayered membranes). We used eq 1 to convert application-specific membrane performance data into permeabilities. We attach the superscript *U*, to denote that these permeabilities have a “universal” definition across applications. Detailed criteria for data selection and descriptions of the conversion, aggregation, and analysis procedures are provided in the SI, along with an .xlsx file containing the complete data set.

We keep  on the left side of eq 1 to facilitate membrane selectivity analysis. The degree to which a membrane separates two solutes increases when the concentration-normalized flux of the permeating solute is large compared to the concentration-normalized flux of the rejected solute. For example, if solutes A and B are acted upon by the same electrochemical driving force but have a concentration ratio of 10:1 within the external fluid, the flux of A through a nonselective membrane will be 10 times that of B because A is 10 times more abundant. Hence, a straightforward way to quantify the separation of two solutes is a separation factor, Γ^*U*^, defined as the ratio of concentration-normalized fluxes:
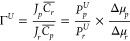
2where the subscripts *p* and *r* refer to the permeating and rejected solutes, respectively. In the limit of a complete separation, the transmembrane flux of the rejected solute would be zero and Γ^*U*^ → ∞.

Inspection of eq 2 reveals that separation is possible when there is a difference in the membrane permeability to the solutes (*P*_*i*_^*U*^), the driving force acting on the solutes , or both. These are the primary “levers” by which separation performance can be controlled. We consider each as we analyze our data set in the following sections.

## 3 Contribution of Membrane Material to Separation Processes

**Figure 1:**
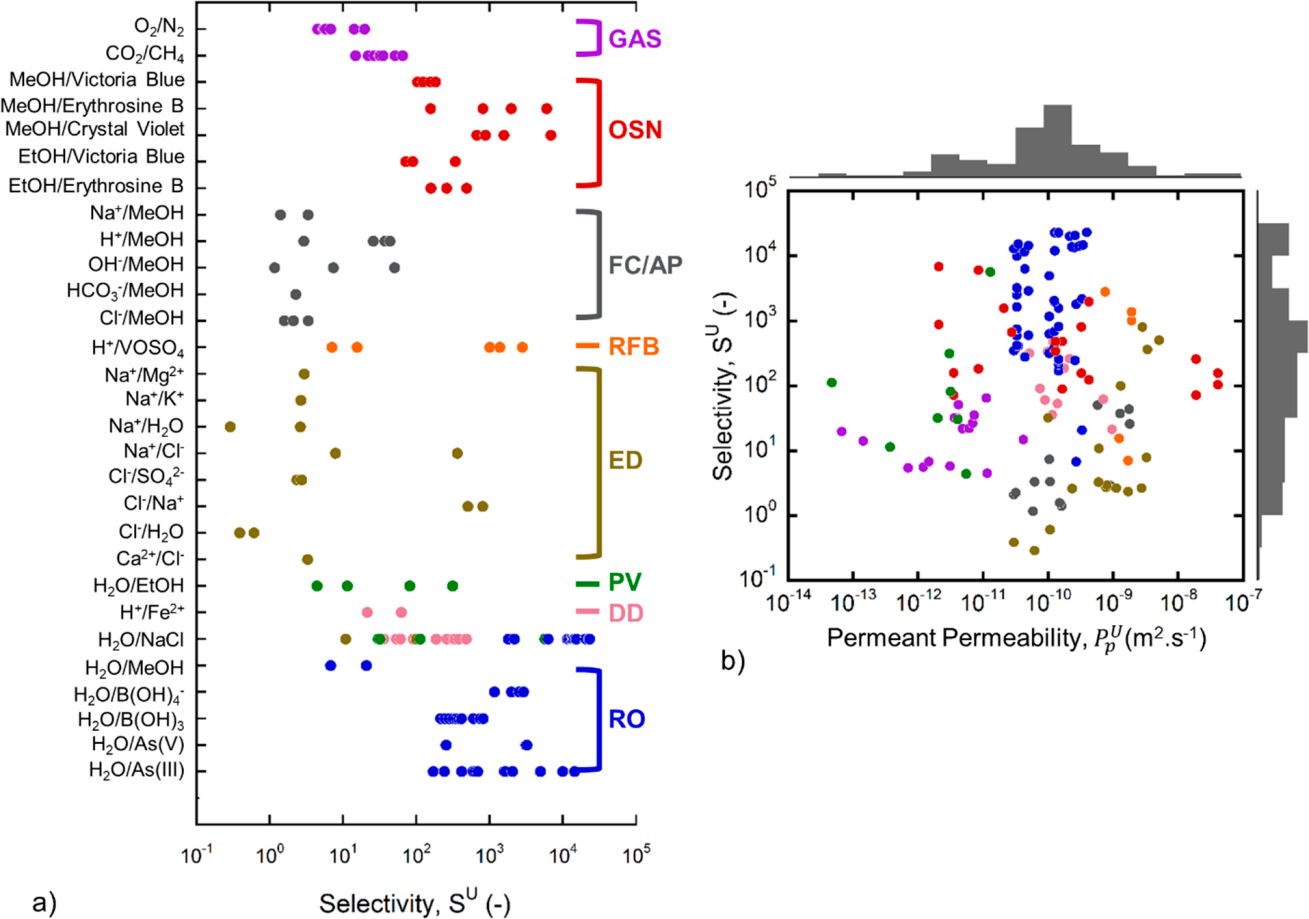
Membrane selectivity (eq 3) categorized by (a) separation and application and (b) the universal permeability to the permeating solute for various applications. Colors denote application as indicated on the right side of panel (a) as defined in Table 1. The histograms in panel (b) represent the distributions of permeabilities and selectivities. Both histograms are scaled to the same axis limit, so the lengths of the bars are directly comparable (also see Figure S3). Please refer to SI (Sections S4–S6) for experimental conditions, assumptions, and application-specific details for each universal permeability calculation.

We begin by considering the role of the membrane material. A key material property is the membrane selectivity, *S*^*U*^, defined as the ratio of solute permeabilities, i.e., the first factor in eq 2:
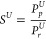
3The selectivity is independent of membrane thickness and driving force and, hence, is an ideal metric for comparing membrane material performance across diverse applications.^35,36^Figure 1a presents the selectivities of all membranes in our data set, organized by row according to separation (i.e., solute pair) and grouped by application (indicated by color). The highest selectivities (>1000) are generally achieved by RO or OSN membranes when water or methanol (MeOH) is the permeating solute. Gas separation membranes achieve intermediate selectivities, while membranes that permeate ions (e.g., for FC, AP, ED, or RFB membranes) tend to have the lowest selectivities (<10).

Figure 1b, inspired by Robeson plots commonly used in gas separation,^37,38^ illustrates the relationship between selectivity and the permeability to the permeating solute. Unlike a typical Robeson plot, however, Figure 1b includes data from many different solutes and separations; therefore, we do not seek to identify a universal “upper bound” on membrane performance because such performance limits depend heavily on solute properties. Instead, we use Figure 1b to compare the permeability and selectivity beyond a single separation. Here, we observe that membranes with the highest selectivity (∼10^4^) have the same permeant permeability (∼10 ^–10^ m^2^ ·s^–1^) as membranes with the lowest selectivity (∼1). By eq 3, this result suggests that membranes achieve high selectivity not by rapidly permeating the permeant solute, but rather by minimizing transport of the rejected solute.

The importance of solute rejection is also conveyed by the data *distribution*, illustrated by the histograms opposite the horizontal and vertical axes. The histograms reveal that there is a broader distribution in selectivity (with many values ranging from 10^1^–10^5^) than in permeant permeability (where most values fall within approximately 1 order of magnitude between 5 × 10^–10^ and 5 × 10^–9^ m^2^·s^–1^). The narrow distribution of *P*_*p*_^*U*^ relative to the broad distribution of *S*^*U*^ implies a broad range of *P*_*r*_^*U*^ values (; eq 3), suggesting that high rejections are a primary driver of high selectivities. Indeed, the interquartile range (middle 50% of all data points) for *P*_*r*_^*U*^ (Figure S3) is 1.1 × 10^–11^–4.1 × 10^–14^ m^2^·s^–1^, much larger than the interquartile range for *P*_*p*_^*U*^ (3.0 × 10^–11^–3.1 × 10^–10^ m^2^·s^–1^). As such, the data of Figure 1 suggest that high selectivity should be attributed in large part to high solute *rejection* rather than high *permeability*. A similar conclusion was articulated previously for RO membranes, where increasing rejection (i.e., decreasing *P*_*r*_^*U*^) would be more likely to reduce the cost of water desalination than increasing water permeability (*P*_*p*_^*U*^).^39^ Our data suggest that this strategy may apply to other separations as well.

### 3.1 Origins of Membrane Selectivity

Membranes achieve selective transport by exploiting differences in the permeating and rejected solutes and the ways in which they interact with the membrane polymer. Such differences are related to the physicochemical properties of the solutes, such as size, charge, or chemical characteristics. We now examine the selectivity data of Figure 1 in light of each of these aspects.

#### 3.1.1 Size-Based Selectivity

Solutes diffuse through dense polymers by moving into transient void spaces (i.e., “free volume elements”) resulting from polymer chain dynamics.^17,40−42^ Since smaller voids form more frequently than larger ones, smaller solutes diffuse more rapidly than larger solutes.^41,43−46^ As such, the selectivity between two solutes is usually expected to increase with increasing difference in size,^9,41,47^ and this physical picture has long been used to rationalize membrane performance in the gas,^48^ nanofiltration,^49^ and RO^42^ literature. Geometric factors such as solute shape can also affect selectivity to an extent.^50^ We do not explicitly account for such factors; however, the hydrated (Stokes) or kinetic radii (Table S2) we use to represent effective size are based on transport measurements that implicitly reflect the irregular shape of nonspherical solutes.

**Figure 2:**
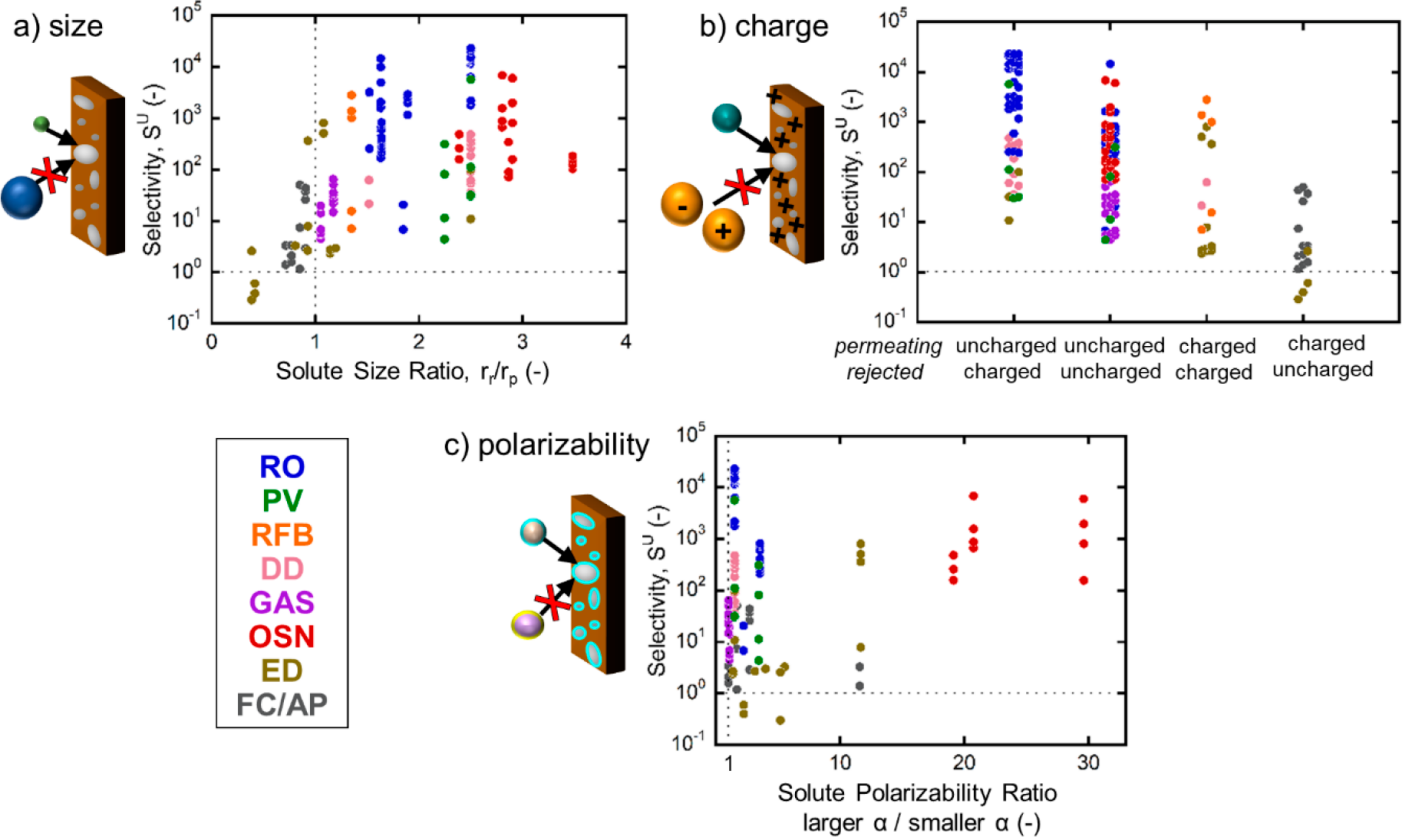
Membrane selectivity by rejection mechanism: (a) solute size (r) ratio, (b) difference in solute charge (permeating solute listed above), and (c) solute polarizability (α) ratio. Subscripts r and p refer to the rejected and permeating solutes, respectively. The ratio of the effective solute diameters (panel (a)) is approximated via the hydrated (Stokes) or kinetic radius (see Table S2). In panel (b), weak electrolytes and salts are considered charged if they are likely to dissociate at the typical process pH, and ions are assigned the charge of the dominant solute at that pH. Solute polarizability is listed in Table S3.

Figure 2a demonstrates that membrane selectivity generally increases with an increasing size ratio, consistent with expectations based on previous reports and the physical picture of free volume elements. However, it is noteworthy that several ion-exchange membranes are selective to solute pairs where the solute size ratio is unfavorable. This result would be impossible if solute size selectivity were the only contributor to selectivity, illustrating the importance of additional mechanisms, such as charge preferential sorption. These additional mechanisms are leveraged by rubbery polymer membranes (i.e., polymers above their glass transition temperature), in which the increased polymer chain mobility can dampen the relative importance of size selectivity and even enable selectivity trends contrary to what is expected based on solute size.^9,51^

#### 3.1.2 Charge-Based Selectivity

Membranes used for liquid-phase separations commonly feature charged functional groups that preferentially sorb solutes of opposite charge (“counterions”) and repel solutes of like charge (“co-ions”) via Donnan exclusion.^52,53^ Neutral (uncharged) solutes are relatively unaffected.^54^ When there is no electric current, electrostatic rejection of the co-ion also causes rejection of the counterion (and hence the entire salt) to maintain electroneutrality.^55,56^ When there is a net electric current, selective transport of counterions occurs because they are preferentially sorbed due to the membrane charge, giving them a much higher concentration than co-ions.^53,55^ Hence, for many given separations, the charges of the permeating and rejected solutes have significant implications for membrane selectivity.

Figure 2b categorizes separations from Figure 1 by the charges of the permeating and rejected solutes. Separations in the uncharged/charged category, such as water desalination by RO or PV, permeate the uncharged solute (e.g., water) and reject the charged solute (e.g., NaCl = Na^+^ and Cl^–^). The membrane charge enhances the performance of uncharged/charged separations that are driven by pressure, which is a major reason this category features higher selectivity than the others. For example, RO achieves higher selectivities than OSN even though OSN generally has larger solute size ratios (Figure 2a) because RO leverages both size and charge-based selectivity. By contrast, OSN separations generally do not involve charged solutes and hence cannot leverage this additional mechanism. As a result, uncharged/uncharged selectivities are, in aggregate, lower (Figure 2b).

Processes in the charged/charged category, including ED, DD, and RFB, separate ions. This category can be further differentiated into separation of oppositely charged ions (“charge selectivity”), separation of different valences with like charge (e.g., monovalent from multivalent cations, “valence selectivity”), and separation of ions with identical charge (e.g., Na^+^ from K^+^, “specific ion selectivity”).^57^ In charge-selective separations (such as ED), transport through the membrane need not be electroneutral due to the presence of an electric potential, and therefore Donnan exclusion can be used to separate oppositely charged solutes (e.g., Na^+^/Cl^–^) with high selectivities (∼1,000; Figure 2b).

By contrast, valence and specific ion selectivities^58−61^ are rarely greater than 50, in large part because they cannot leverage the Donnan exclusion mechanism. A noteworthy exception is the separation of H^+^ from like-charged redox species in RFB, which has a high selectivity (∼1,000; Figure 2b) due to the extremely high mobility of protons compared to other ions. Outside of separations that permeate H^+^, engineering membranes with specific ion and valence-selectivity remain among the most challenging research problems in membrane science.^57,62^

Finally, separations in the charged/uncharged category permeate charged solutes (e.g., OH^–^) and reject uncharged solutes (e.g., MeOH). These separations are commonly encountered in FC and AP devices employing polymer electrolyte membranes (i.e., ion exchange membranes). Here, charge (i.e., Donnan exclusion) is not a viable rejection mechanism, because the rejected solute is neutral. Furthermore, many separations relevant to FC/AP applications involve an unfavorable size ratio (Figure 2a) because the permeating solute is a hydrated ion with a radius at least as large as that of the rejected solute. As a result, this category exhibits the lowest selectivities of the four presented in Figure 2b. In spite of the unfavorable size ratio and charge state, however, selectivities greater than unity are still observed. This suggests that exceptionally high sorption of counterions via electrostatic attraction^52^ may enable charged membranes to selectively transport counterions even when the neutral solutes are smaller in (hydrated) size, further underscoring the importance of membrane–solute interactions in charge-based selectivity.

#### 3.1.3 Selectivity beyond Size and Charge: Nonelectrostatic Chemical Effects

Beyond size and charge, a variety of factors broadly related to the chemical properties of the solutes can also affect transport and selectivity. For example, polarity, polarizability, hydrogen bond donor and acceptor functionality, hydrophobicity/hydrophilicity, and van der Waals interactions have all been recognized as factors that influence solute transport in certain cases.^37,46,63−69^ The dielectric ion exclusion mechanism, recognized in RO and NF membranes, is related (via the dielectric constant) to the microscopic dipole moments of solutes and polymer chains,^70^ and the condensability (or critical temperature) of gas molecules is known to control sorption selectivity in rubbery polymers.^9^

For the purposes of this analysis, we adopted solute polarizability as a metric for quantifying differences in solute chemistry (see Table S3). Polarizability describes how easily a solute’s electron cloud can be distorted from its usual shape by the presence of an electric field or charge and has been correlated with several relevant interactions, including the strength of ion binding to charged sites in ion exchange membranes,^71^ van der Waals interactions that give rise to ion-specific effects in membranes and to “Hofmeister effects” in biological systems,^72^ and the octanol–water partition coefficient of neutral molecules.^46,68,69^

Figure 2c organizes the selectivity data from Figure 1 according to the ratio of solute polarizabilities, where a larger ratio indicates a greater contrast in the polarizabilities of the solutes. There is no clear relationship between selectivity and polarizability ratio: near a ratio of 1, where there is little contrast in permeating and rejected solute polarizability, both very low and very high selectivities are observed. High selectivities are observed when the polarizability ratio is large (e.g., some OSN and ED separations); however, for these solute pairs, there is also either a large contrast in solute size (for OSN) or charge (for ED).

Altogether, the data in Figure 2 illustrate that the selectivity of current commercial membranes across all applications can effectively leverage size and charge differences within the solute pair, and in some cases (such as RO) these mechanisms work synergistically. On the other hand, differences in solute polarizability appear to have a much smaller influence on membrane selectivity. This suggests that development of membranes that could better leverage differences in polarizability (or other nonelectrostatic chemical effects) could enable substantial improvements in selectivity for specific ions and for applications involving neutral solutes (e.g., uncharged/charged separations), either by inducing preferential sorption or by hindering the transport of the target solute. Gains by selective mechanisms other than size would have the greatest impact in rubbery polymers.

## 4 Contribution of Driving Forces to Separation Processes

Besides the selectivity of the membrane material, the driving force (i.e., the gradient in electrochemical potential, ) is another factor that can be tuned to improve separation (see eq 2). Pressure, concentration, and electric potential all contribute to the electrochemical driving force (eq S1), and while they are often applied uniformly to the gas or liquid mixture treated by the membrane, they may affect some solutes differently than others. For example, an applied electric field motivates the transport of charged solutes but not that of neutral solutes (neglecting electro-osmosis), potentially enhancing the separation factor between such solutes.

**Figure 3:**
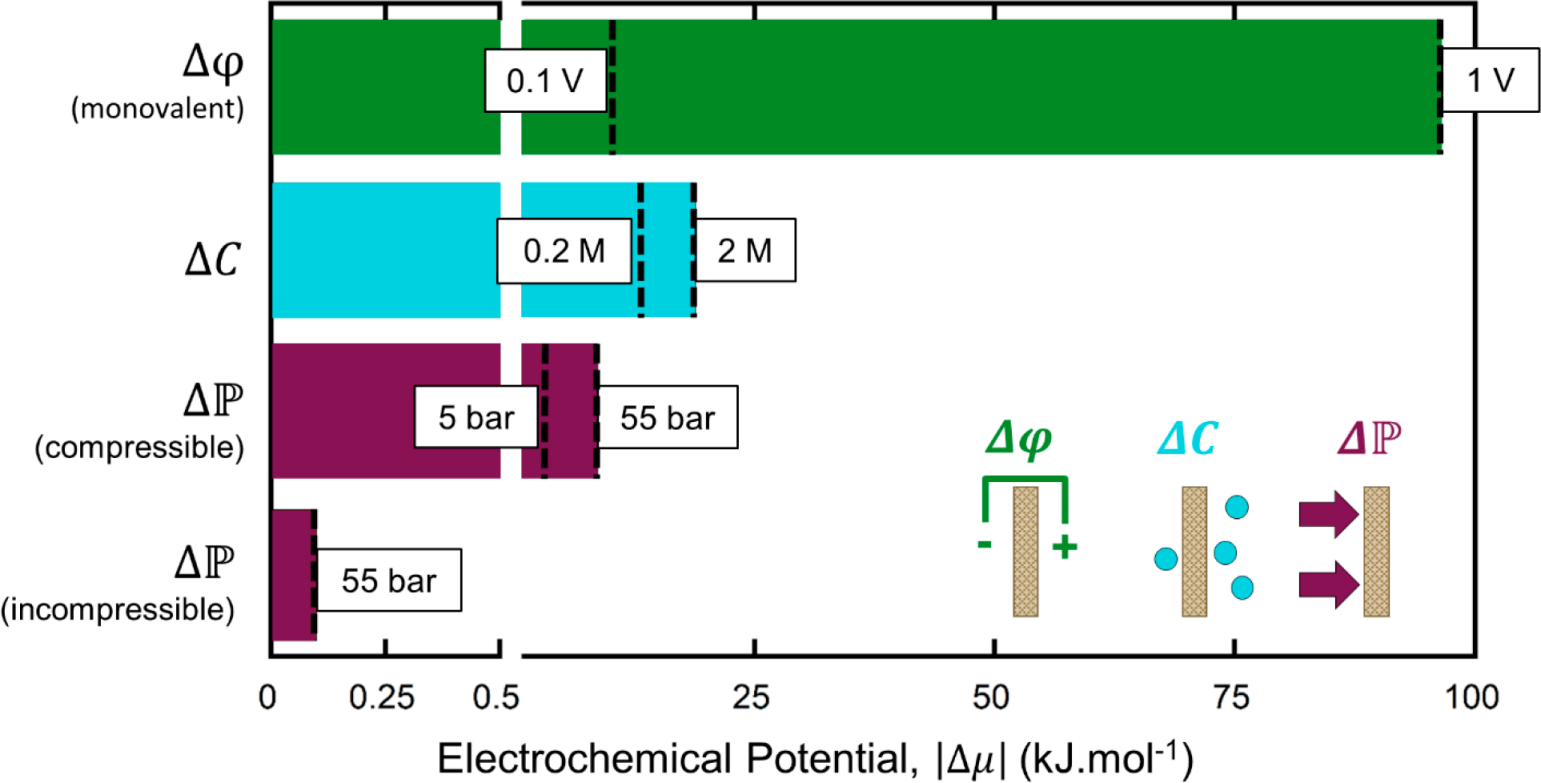
Electrochemical potential produced by typical process conditions used in several industrial separations. Δφ: a monovalent solute under an applied potential of 1 V, typical of ED;^74,75^ Δ*C*: a concentration difference of 2 M, with a downstream concentration of 0.001 M, typical in direct methanol FC;^73,76^, compressible: gas at a transmembrane pressure of 55 bar, typical of industrial GAS separations;^77^, incompressible: water at a transmembrane pressure of 55 bar, typical of RO.^78^ All values were calculated at 25 °C.

In Figure 3, we convert pressure, concentration, and electrical driving forces into electrochemical potential using eq S1. The spans of the bars represent the ranges of typical membrane process conditions for each. Strikingly, the electrochemical potential created by applying a 1 V potential to a monovalent ion (as in ED) is 4 orders of magnitude larger than that generated by a pressure of 55 bar applied to liquid water (an incompressible fluid; compare top and bottom bars) as in RO. Stated differently, a pressure of more than 54,000 bar would be required to achieve the same electrochemical potential on water as that experienced by the ion in the electric field. Therefore, application of electricity offers an enormous opportunity to enhance selectivity in separations that involve charged solutes, even if the membrane material itself has poor selectivity.

Additionally, a typical concentration difference (2 M upstream and 0.001 M downstream, as in fuel cells) creates an electrochemical potential more than an order of magnitude larger than that of 55 bar pressure applied to an incompressible fluid (as in RO); see Figure 3. Concentration gradients are present in most membrane processes as a consequence of the separation (as opposed to being intentionally applied; although DD is an exception). As such, they usually oppose the (pressure or electric) driving force applied to the permeating solute. For the rejected solute, the induced concentration gradient can cause undesirable transport through the membrane (e.g., salt diffusion in RO,^43^ MeOH crossover in direct methanol fuel cells^73^).

The chemical potential associated with a concentration gradient is related to the logarithm of the ratio of the downstream to upstream (feed) concentrations (eq S1), For example, a 2 M difference in concentration given a downstream concentration of 0.001 M (as in the case of MeOH in fuel cells) produces an orders-of-magnitude larger driving force than a 2 M difference in concentration given a downstream concentration of 53.7 M (as in the case of water in RO) since . An additional consequence of this logarithmic dependence is that reducing the *higher* solute concentration does little to reduce the driving force. For example, a 10-fold decrease in the higher concentration (e.g., 2 to 0.2 M) with a constant lower concentration (e.g., at 1 mM) decreases the electrochemical potential by only 32% (from 19 to 13 kJ·mol^–1^, Figure 3).

Given the small magnitude of the incompressible fluid pressure driving force relative to the concentration or electric potential driving forces, the dominance of pressure-driven liquid processes such as RO may seem surprising. However, its high overall separation performance can be understood by considering the interplay among driving force, membrane thickness, and material selectivity, which we discuss in the next section.

## 5 Applying the Framework: Material Selectivity and Driving Forces in Concert

So far, we have discussed how membrane properties and driving forces individually affect separations. We now examine how these factors contribute to the overall process selectivity via three case studies. In doing so, we illustrate how the physicochemical properties of the solute pair impose constraints on the available driving forces and selectivity mechanisms and thereby dictate what improvements are most likely to improve overall separation performance.

**Figure 4:**
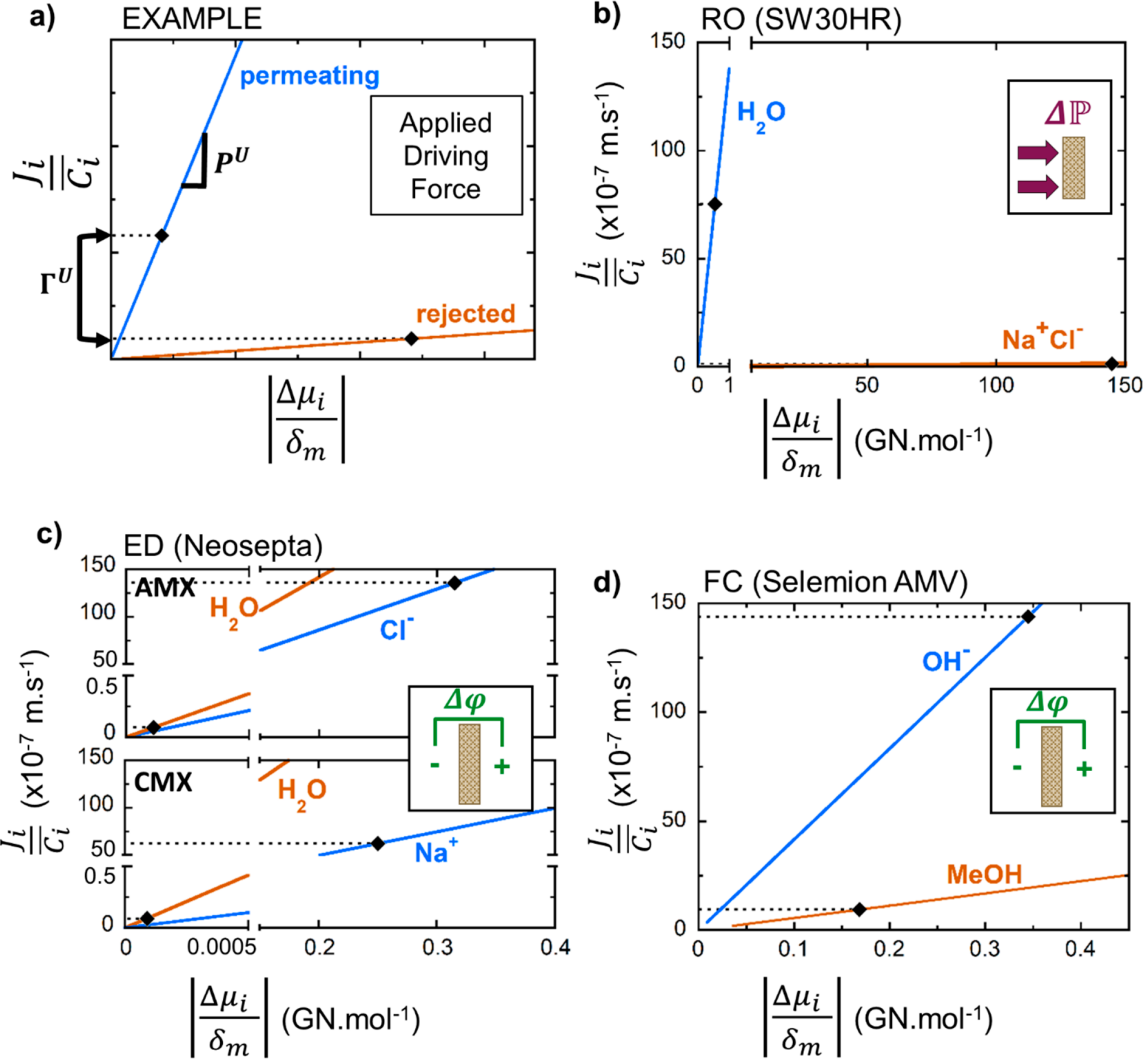
Graphical illustration of eq 1 for (b) RO, (c) ED, and (d) FC applications, wherein the vertical axis represents the concentration-normalized flux, , of a solute, and the horizontal axis represents the molar electrochemical potential driving force acting on it, . The slopes of the lines are the membrane permeability, *P*_*i*_^*U*^, to the permeating (blue) and rejected (orange) solutes. The black diamonds are typical process conditions for each application. (a) An annotated example plot. (b) The flux of water and NaCl through SW30HR in RO (55 bar; 32,000 ppm of NaCl, 99.7% rejection).^78^ (c) The flux of water, Na^+^, and Cl^–^ through Neosepta AMX and CMX in ED (12,000 ppm/1,200 ppm of NaCl, 0.5 V applied potential).^4,6,6^ (d) The flux of MeOH and OH^–^ through Selemion AMV in a fuel cell (2 M/0.001 M MeOH fuel, 0.4 V applied potential).^76,79^

We illustrate the case studies through a graphical representation of eq 1 (Figure 4), in which we plot the concentration-normalized flux of each solute (, vertical axis) as a function of the driving force acting on it (, horizontal axis). The slopes of the blue and orange lines indicate the permeability of the membrane (*P*_*i*_^*U*^) to the permeating and rejected solutes, respectively. Highly rejected solutes have a slope close to 0 (horizontal), and the greater the difference in slope between the permeating and rejected solute lines, the greater the selectivity of the membrane (eq 3). Note that membrane thickness only impacts the flux and not the selectivity (see eqs 1 and 2). For each solute, a black diamond represents the typical process conditions and corresponding flux. A table of these metrics (*S*^*U*^, Γ^*U*^, *P*_*i*_^*U*^, etc.) for each case study is provided in Table S5.

### 5.1 Case Study 1: Reverse Osmosis (RO) Desalination

RO (Figure 4b) accounts for 80% of the seawater and brackish water desalination applications,^28^ producing over 100 million m^3^ of clean water per day globally.^80^ In this pressure-driven uncharged/charged separation, water is the permeating solute and NaCl (dissociated as Na^+^ and Cl^–^ ions) is the rejected solute. The RO membrane material is highly permeable to water (steep slope) and nearly impermeable to salt (horizontal slope) due to the favorable combination of size and charge selectivity already discussed. Application of pressure to an incompressible fluid such as water produces a very small driving force compared to the NaCl concentration gradient established across the membrane, resulting in a much smaller horizontal axis value for water than NaCl. RO is able to produce useful flux in spite of the low driving force because it employs an exceptionally thin (on the order of 100 nm^81^) active layer, which increases chemical potential gradient  (see eq 1). Employing such a thin membrane is only feasible in this case because of the membrane’s high selectivity. Increasing the gradient by reducing membrane thickness (i.e., shifting right along the horizontal axis of Figure 4b) increases the flux of water dramatically due to the steep slope (high permeability) but—crucially—does not result in substantially greater NaCl flux due to its low permeability.

### 5.2 Case Study 2: Electrodialysis (ED) Desalination

ED (Figure 4c) is an alternative, electrically driven desalination process that has comparable cost and energy efficiency to RO for brackish water treatment.^28,30,82^ It accomplishes desalination by *removing ions* rather than permeating water through a membrane (as in RO), and therefore high water permeation is detrimental to process efficiency.^83−86^ As such, ED utilizes a pair of charged membranes to electrostatically separate oppositely charged ions (e.g., one membrane permeates Na^+^ and rejects Cl^–^, and the other does the opposite; Figure 2b). In general, the selectivity of these membranes for counterions over co-ions is high (Table S5); however, the permeability to water is greater than that to the ions (as indicated by the steeper slope of water) for both membranes,^87^ meaning that their ion/water selectivities are lower than unity.

Because the membranes are highly permeable to water, it would be impossible to perform desalination in ED if water were subjected to the same electrochemical driving force as the ions. However, the applied electric potential acts only on the ions. Neglecting electro-osmosis, the only driving force for water permeation is the difference in osmotic pressure between the feed and concentrate sides of the membrane (which is really a difference in water concentration). The orders-of-magnitude difference in these driving forces (horizontal axis values in Figure 4c; see also Figure 3) makes effective separation (Γ^*u*^ > 1) of ions from water possible, even though *S*^*U*^ < 1 (see eqs 2 and 3). In fact, the counterion/water separation factor in this brackish water ED example is approximately 30 times greater than the water/salt separation factor in seawater RO (Table S5). Thus, whereas RO is enabled by high membrane selectivity, ED is enabled by the application of an electrical driving force which only acts on the permeating solutes. This also explains why ED is most often used when the driving force on water is low such as brackish water desalination. Efficiently treating high-salinity gradients with ED will require mitigating the water flux resulting from the higher osmotic pressure (i.e., a shift right on the horizontal axis, Figure 4c)^31,84,88^ by reducing the membrane permeability to water (i.e., a shallower slope, Figure 4c) or using creative process designs, such as applying pressure^89^ or introducing a neutral “osmotic ballast”^85^ to counteract the osmotic driving force. Finally, note that because of the poor selectivity of ion exchange membranes, reducing the membrane thickness would increase the fluxes of both water and ions, compromising the rejection of water. For this reason, ion exchange membranes for ED and FC have an optimal thickness of tens^90^ to hundreds^91^ of micrometers that balances higher permeability to counterions against lower rejection.

### 5.3 Case Study 3: Alkaline Direct Methanol Fuel Cells (ADMFCs)

Alkaline direct methanol fuel cells have potential as portable energy sources due to their ease of transport and the high energy density of methanol.^92−95^ These fuel cells require a polymer electrolyte membrane between the anode and the cathode to permeate OH^–^ while blocking the crossover of MeOH, which limits cell efficiency.^23,94−96^ The permeating OH^–^ is larger than the MeOH, making this a highly challenging charged/uncharged separation with an unfavorable size ratio of 0.85 (see Figure 2a,b).

The ADMFC leverages both material selectivity and solute-specific driving forces (Figure 4d). The membrane is selective (*S*^*U*^ > 1) for OH^–^, as indicated by its steeper slope, and the electric driving force acts only on the permeating OH^–^, as indicated by its larger horizontal axis value compared to that of MeOH. Despite the use of both “levers” to achieve this separation, however, FC performance is still limited by the undesirable crossover of MeOH. Increasing the thickness of the membrane or decreasing the MeOH concentration to reduce the chemical potential gradient for MeOH crossover either has a greater (undesirable) effect on the OH^–^ flux^23,97^ or reduces the energy density of the fuel to an undesirable extent.^23,95^ Hence, research efforts to decrease the membrane permeability to MeOH are still needed. Considering the challenging constraints imposed by the properties of the OH^–^/MeOH solute pair (e.g., charged/uncharged and unfavorable size ratio), such efforts should prioritize approaches that exploit contrasts in other chemical properties. For example, the introduction of selectively binding moieties similar to MOFs used in gas separation^98^ or metal binding ligands added to ion exchange membranes^99^ may provide a way to target the effects of membrane modifications to OH^–^.

Taken together, these case studies illustrate how membrane material properties (i.e., selectivity), process conditions (i.e., driving forces), and physichochemical constraints (i.e., solute properties) converge to determine overall separation performance, and that a common conceptual framework (encapsulated in Figure 4 and eq 1) can be used to understand their interactions, regardless of the specific application.

## 6 Summary and Outlook

In summary, we have examined membrane performance across a variety of separations (solute pairs) to identify a conceptual framework by which to understand the capabilities of state-of-the-art membrane processes. Specifically, we have observed that

- High selectivity is usually promoted by good *rejection* rather than fast *permeation*.
- Current membranes rely primarily on size and charge to achieve high selectivity.
- Reducing membrane thickness benefits process performance when the membrane material is highly selective.
- Applying driving forces that act only on specific solutes (e.g., electric potential which acts on ions but not neutral molecules) is a potent strategy for overcoming poor material selectivity to achieve good separation factors.

Informed by these observations, we suggest three broad research themes that have the potential to positively impact many different applications and hence present ripe opportunities for cross-pollination: (1) increasing the precision of size selectivity, (2) advancing chemistry-based selectivity mechanisms, and (3) investigating new ways to apply solute-specific driving forces.

Given the predominance of size selectivity among current materials (see Figure 2a), further improvement in this mechanism would have a significant impact. Current fabrication methods usually produce membranes with a wide distribution in the size and shape of free volume elements through which solutes permeate (i.e., nonuniform “pore size”). More precise control over the membrane morphology, and specifically a narrower distribution of free volume element size, has been shown to improve selectivity in gas^100^ and liquid^101^ separation membranes. Emerging approaches to achieve exquisite selectivity include mixed-matrix membranes containing metal or covalent organic frameworks (MOFs or COFs),^102^ polymers of intrinsic microporosity (PIMs),^103−106^ molecular imprinting,^107,108^ crystallinity,^109,110^ zwitterionic microchannels,^111^ and additive manufacturing.^112^ Active research in this area spans applications as diverse as lithium recovery from water,^102^ small molecule separation,^113^ direct air capture of CO_2_,^114,115^ redox flow batteries,^103−106^ gas separations,^107,108^ and fuel cell membranes,^109,110^ indicating that it is of high interest throughout the membrane community.

A second major challenge common to multiple applications is the need for additional mechanisms (beyond size and charge) for differentiating solutes (see Case Study 3). We note that many current membranes do not exploit contrasts in solute polarizability (a proxy for nonelectrostatic chemical properties). Advancements in this area will require progress in two major areas. First, there is a need to identify additional solute descriptors that capture aspects of solute chemistry, such as hydration state, binding affinity toward different functional groups, etc. For example, a diverse array of specific-ion (“Hofmeister”) effects were recently correlated to a metric derived from site-specific charge density.^116^ In general, we see great potential for computational methods to inform this area. Empirical methods such as classical molecular dynamics (MD) can generate rich insights into transport^117−120^ and ion solvation,^121,122^ while first-principles methods including density functional theory (DFT) and *ab initio* MD (AIMD) can estimate many relevant phenomena involving short- and medium-range chemical effects, such as energy barriers for diffusion, charge distribution, or binding affinity^123−129^ without relying on empirically fitted force fields. Depending on the solute and property of interest, advanced characterization methods such as ambient pressure X-ray photoelectron spectroscopy (XPS), grazing incidence small-angle X-ray scattering (GISAXS), or scanning electrochemical microscopy (SECM) can also enrich our understanding of solute properties and solute–membrane interactions.^130,131^

A greater understanding of contrasts in solute chemistry, including nonelectrostatic effects, could also support the development of membranes with highly specific solute–membrane interactions. This is another area where innovative approaches in different applications might inspire related developments in others. Leading efforts in liquid separations include introducing functionalities that selectively bind a particular solute (e.g., “host–guest chemistry” or “ion capture”)^99,132,133^ and bioinspired moieties inspired by biological membranes, such as aquaporins.^74,134^ The gas separation community has long known that rubbery polymers can exploit differences in gas condensability to achieve selective sorption and has more recently explored constituents such as silver ions and MOFs to enhance the sorption of specific solutes in order to separate olefins from paraffins.^135−137^ Carrier facilitated transport, in which a selective, mobile carrier binds to a target solute, diffuses across the membrane, and releases it on the permeate side, has been studied in both gas and liquid separations for decades, but although this approach can achieve exceptional selectivities, it remains difficult to scale beyond the laboratory.^9^

Finally, there is considerable potential for the application of solute-specific driving forces to enhance separation performance, especially in the case of the selective removal of dilute solutes from complex mixtures, in which the concentration gradient against transport can be quite significant. To date, this strategy is largely limited to applying electric fields in charged/uncharged or charged/charged separations, but a variety of new and creative strategies could further expand it. For example, a few recent studies have examined combinations of driving forces, such as pressure with electric potential^89^ or a phase change.^138^ Concentration gradients could be mitigated by selective precipitation of a solute out of the downstream fluid,^139^ and pH changes can be used to “activate” electric and electrostatic mechanisms by changing the charge state of weakly dissociated solutes, as practiced in RO boron removal.^140,141^ Size exclusion can be enhanced by placing bulky, selectively binding ligands into the feed solution, and by exotic driving forces, such as the Soret effect used in isotope separations.^142^

Although the analysis presented here is highly simplified, we believe this conceptual framework will provide an effective vehicle for identifying opportunities for knowledge transfer among membrane subdisciplines. By recognizing common challenges, we hope that membrane researchers will make connections and draw inspiration from outside their respective fields, thereby accelerating membrane innovation.

## Supporting Information Available

List of symbols and unit conversions; solute size and permeability data; derivation of universal permeability equation; conversions between typical figures of merit; notes on compilation and conversion of membrane transport data; additional calculation details supporting Figures 3 and 4; histograms of solute permeability data; and description of tabulated membrane performance data file (PDF)
Tabulated membrane performance data (XLSX)
