# Supplementary material for: Unifying the Conversation: Membrane Separation Performance in Energy, Water, and Industrial Applications
Source: ACS ES T Eng. 2024 Jan 26;4(2):277–89. doi: 10.1021/acsestengg.3c00475 (PMC10862477; doi:10.1021/acsestengg.3c00475)
Supplement: Supplementary file 1 — ee3c00475_si_001.pdf [file ee3c00475_si_001.pdf]

Supporting Information

Unifying the conversation: Membrane Separation Performance in Energy, Water, and Industrial Applications

Sarah M. Dischinger[a], Daniel J. Miller[a,*], David A. Vermaas[b,*], Ryan S. Kingsbury, [c,d*]

[a] Chemical Sciences Division, Lawrence Berkeley National Laboratory, Berkeley, CA 94720, USA
[b] Department of Chemical Engineering, Delft University of Technology, 2629HZ, Delft, The Netherlands
[c] Energy Storage and Distributed Resources Division, Lawrence Berkeley National Laboratory, Berkeley, CA 94720, USA
[d] Department of Civil and Environmental Engineering and the Andlinger Center for Energy and the Environment, Princeton University, Princeton, NJ 08540, USA

[*] Corresponding authors: Correspondence to Ryan S. Kingsbury (kingsbury@princeton.edu), David A. Vermaas (D.A.Vermaas@tudelft.nl) and Daniel J. Miller (danieljosephlangmiller@gmail.com)

Contents

Section S1. Search terms, list of symbols and unit conversions 2
Section S2. Species size and polarizability 3
Species Size 3
Solute Polarizability 5
Section S3. Derivation of universal permeability 6
Section S4. Conversions between typical figures of merit and universal permeability 10
Section S5. Compilation of membrane transport data 12
Scope of data considered 12
Calculation of electrochemical potential difference 13
Calculation of universal permeability P^U 14
Section S6. Converting common driving forces to electrochemical potential 20
Section S7. Developing concentration-normalized flux vs. driving force plots 22
Section S8. Description of tabulated membrane performance data 25
Section S9. Histograms of Permeability Data 28
References 29

Section S1. Search terms, list of symbols and unit conversions

The interest in membrane technology was rated by counting the scientific publications and patents in the last decade, using ISI Web of Science and Google Patents, respectively. Search terms: "reverse osmosis" OR "gas separation" OR "ion exchange membrane" OR "fuel cells" OR "redox flow battery" OR "diffusion dialysis" OR "pervaporation" OR "nanofiltration" OR "diffusion dialysis"

The estimated annual rate of technological improvement in membrane separations is based on search results from Technology Search Portal, http://technologyrates.mit.edu/. Search term: "membrane", Domain_ID 210B01D. The technology improvement rate is based on models developed through analysis of US patents and citations within patents. These models were validated against empirical studies of 30 technologies, and then projected to other technologies.

Table S1. List of symbols

Term	Symbol	Units
Charge	z	dimensionless
Concentration	C	mol.L^{-1}
Conductivity	κ	S.cm^{-1}
Diffusion coefficient	D	m^{2}.s^{-1}
Electric current density	I	A.m^{-2}
Electric potential	φ	volts
Electrochemical potential	μ	kJ.mol^{-1}
Faraday's constant	F	96485 C.mol^{-1}
Ideal gas constant	R	8.314 J.mol^{-1}.K^{-1}
Membrane (selective layer) thickness	δ_m	µm
Molar flux	J	mol .m^{-2}.s^{-1}
Molar scale activity coefficient	γ^M	dimensionless
Molar volume	V_i^m	cm^{3}.mol^{-1}
Mole fraction	x	dimensionless
Mole of solute	n	mol
Partition or sorption coefficient	K	dimensionless
Permeability, concentration-based	P	cm^{2}.s^{-1}
Permeability, pressure-based (for gas)	P_i^G	Barrer
Pressure, osmotic	π	bar
Pressure, partial	$\mathbb{P}_i$	bar
Pressure, total	$\mathbb{P}$	bar
Pervaporation separation factor of solute i over solute j	$\vartheta_{i/j}$	dimensionless
Rejection	Rej	%
Salt permeance	B	L.m^{-2}.hr^{-1}
Selectivity	S	dimensionless
Separation factor	Γ	dimensionless
Temperature	T	K
Transport number	t	dimensionless
Water permeance	A	L.m^{-2}.hr^{-1}.bar^{-1}

**Subscripts and Superscripts**		
Solute index	i	
Water	w	
Upstream (i.e., high electrochemical potential)	u	
Downstream (i.e., low electrochemical potential)	d	
Solution phase	s	
Membrane phase	m	
Permeating	p	
Rejected	r	
Universal	U	

Unit Conversions:

1 Siemens [=] $\frac{6.24 \times 10^{18} electrons}{sec*volt}$

1 Barrer [=] $\frac{10^{-10}\ cm^3\ (STP)*cm}{cm^2*sec*cmHg}$ [=] $4.403 \times 10^{-15} \frac{mol*cm}{cm^2*sec*cmHg}$ [=] $3.3 \times 10^{-16} \frac{mol*meter}{meter^2*sec*Pa}$

Section S2. Species size and polarizability

Species Size

We adopt the effective radius of each solute as a descriptor of its size. Ion size is taken as its hydrated radius because ions are thought to permeate the membrane in a partially- or fully-hydrated state [1,2]. When we were unable to find a directly measured hydrated radius, we estimated it using Avogadro software [3] by adding the radius of a representative solvent probe (water, 1.38 Å) to the largest dimension of the molecular structure. This "molecular size" approximates the hydrated solute size and is analogous to the procedure commonly used to construct the solvent accessible or van der Waals surface of a molecule [4]. In cases where a salt permeates as an ion pair (e.g., NaCl (aq) during RO), the effective radius of the salt was calculated as the geometric mean of the respective hydrated ion radii. The size of an uncharged solute is taken as the Stokes radius, calculated from diffusivity measurements [5]. The size of a gaseous solute is taken as the kinetic radius, which is a widely accepted measure of gas size [6,7].

Table S2. Solute size

Species	Type	Radius (Å)	Ref
Na^{+} (aq)	hydrated	3.58	[8]
Cl^{-} (aq)	hydrated	3.32	[8]
NaCl (aq)	hydrated	3.45	average[a]
K^{+} (aq)	hydrated	3.31	[8]
Li^{+} (aq)	hydrated	3.82	[8]
Mg^{+2} (aq)	hydrated	4.28	[8]
Ca^{+2} (aq)	hydrated	4.12	[8]
Fe^{+2} (aq)	hydrated	4.28	[8]
OH^{-} (aq)	hydrated	3.00	[8]
H_3O^{+} (aq)	hydrated	2.82	[8]
H_2O (l)	kinetic	1.38	[8]
VO^{+2} (aq)	hydrated	3.82	[9]
SO_4^{-2} (aq)	hydrated	3.79	[8]
$VOSO_4$ (aq)	hydrated	3.80	average[a]
As(III) as $H_2AsO_3^{-}$	molecular	2.25	estimated[b]
As(V) as $HAsO_4^{2-}$	hydrated	2.10	[10]
$B(OH)_4^{-}$	Stokes	2.61	[11]
BF_4^{-}	hydrated	3.68	[12]
$B(OH)_3$	molecular	2.26	[13]
Methanol	Stokes	2.55	[14]
Ethanol	Stokes	3.10	[14]
Calcein (similar in structure to erythrosin B)	Stokes	7.40	[15]
Crystal Violet	molecular	7.15	[16]
Victoria Blue	molecular	8.88	estimated[b]
CH_4 (g)	kinetic	3.87	[7]
N_2 (g)	kinetic	3.64	[7]
O_2 (g)	kinetic	3.46	[7]
CO_2 (g)	kinetic	3.30	[7]

[a] Geometric average of the respective ion sizes

[b] Effective molecular size was estimated using Avogadro software [3]

Solute Polarizability

The polarizability of a solute describes how easily its electron cloud can be distorted from its usual shape by the presence of an electric field or charge [5], and carries units of $Å^3$ or $C.m^{-2}.V^{-1}$. Ion polarizability is known to affect the way ions in solutions hydrate and interact with surfaces or other ions [17–20]. For salts that transport as an ion pair, the polarizability of the salt is estimated as the geometric mean of polarizabilities of the individual ions. For uncharged molecules, the polarizability is directly related to optical and vibrational properties that may affect, for example, solubility or interactions with the hydrogen bond network of a solvent [19].

Table S3. Atomic or molecular polarizability ($Å^3$)

Solute	Polarizability ($Å^3$)	Ref
Na^+ (aq)	0.279	21
Cl^- (aq)	3.253	21
NaCl (aq)	0.953	average[a]
K^+ (aq)	0.873	21
Mg^{+2} (aq)	0.07	20
SO_4^{-2} (aq)	4.432	21
Ca^{+2} (aq)	0.588	21
OH^- (aq)	1.91	22
H_3O^+ (aq)	1.19	22
EtOH	5.00	23
MeOH	3.23	23
CH_4 (g)	2.60	23
N_2 (g)	1.76	23
O_2 (g)	1.60	23
H_2O (l)	1.45	23
CO_2 (g)	2.65	23
$B(OH)_3$	5.15	24
Crystal Violet	67.0	25
erythrosine B	95.6	26

[a] Geometric average of the respective ion values

Section S3. Derivation of universal permeability

The driving force for transport of solute i across a membrane is the difference in electrochemical potential of the solute, $\Delta\mu_i$ (kJ.mol^{-1}), between the upstream and downstream sides of the membrane. The electrochemical potential includes contributions from pressure, electric potential, and solute activity (i.e., concentration) [27]:

$$\Delta\mu_i = RT\ ln\left(\frac{\gamma_{i,d}^{M} C_{i,d}}{\gamma_{i,u}^{M} C_{i,u}}\right) + Fz_i(\varphi_d - \varphi_u) + V_i(\mathbb{P}_d - \mathbb{P}_u) \qquad \text{Eqn. S1}$$

where R (8.314 J.mol^{-1}.K^{-1}) is the ideal gas constant, T (K) is the absolute temperature, γ_i (dimensionless) is the molar-scale activity coefficient (corresponding to an infinite dilution reference state), C_i (mol.L^{-1}) is the concentration, F (96485 C.mol^{-1}) is the Faraday constant, z_i is the charge of the solute (including sign), φ (V) is the electric potential, V_i (m^3.mol^{-1}) is the molar volume of the solute, $\mathbb{P}$ (Pa) is the pressure, and subscripts u and d denote the upstream and downstream sides of the membrane, respectively. Here, we define "upstream" and "downstream" with respect to the direction of transport of the solute, i.e., solutes always move from upstream to downstream and the net electrochemical potential is always negative. It is important to note that, by way of the molar volume and charge of the solute, the electrochemical potential is a function of the physicochemical properties of the solute.

As a result of a gradient in electrochemical potential, a net flux will occur. The flux of solute i through a membrane, J_i, is proportional to the electrochemical potential gradient within the membrane, $\frac{\partial\mu_i^m}{\partial y}$, by a factor of the phenomenological constant L [28]:

$$J_i = -L\frac{\partial\mu_i^m}{\partial y} \qquad \text{Eqn. S2}$$

where the positive y-direction is oriented from upstream (high electrochemical potential) to downstream (low electrochemical potential). The physical interpretation of 1/L is that the net driving force for transport is counteracted by a resistivity to transport imposed by the membrane itself. The difference in electrochemical potential across the membrane represents a system that is out of equilibrium, and drives the net transport of solutes. The balance between electrochemical potential difference and resistivity is, by definition, a thermodynamically irreversible process, as the friction associated with transport of solutes causes an entropy increase [27,29]. As we will see later in this section, the resistivity is often quantified as permeability, P_i (m^2.s^{-1}), its inverse. A membrane with a high permeability has a low resistivity to transport, and vice versa.

The electrochemical potential gradient inside the membrane is derived from the definition of the electrochemical potential (Eqn. S1) as:

$$\frac{\partial\mu_i^m}{\partial y} = RT\frac{\partial(\ln(\gamma_i{}^m\, C_i{}^m))}{\partial y} + Fz_i\frac{\partial\varphi^m}{\partial y} + V_i\frac{\partial\mathbb{P}^m}{\partial y} \qquad \text{Eqn. S3}$$

The pressure within a solution-diffusion (i.e., non-porous) membrane is uniform across its thickness (i.e., $\partial\mathbb{P}^m = 0$)[28]. Additionally, as we do not have enough information to calculate the activity coefficient within the membrane, we estimate that the change in the activity coefficient through the membrane is negligible (i.e., $\frac{\partial\gamma^m}{\partial y} = 0$). Therefore, the electrochemical potential gradient within the membrane simplifies to:

$$\frac{\partial \mu_i{}^m}{\partial y} = \frac{RT}{C_i{}^m}\frac{\partial C_i{}^m}{\partial y} + Fz_i\frac{\partial \varphi^m}{\partial y} \qquad \text{Eqn. S4}$$

Eqn. S2 then becomes:

$$J_i = -L\left(\frac{RT}{C_i{}^m}\frac{\partial C_i{}^m}{\partial y} + Fz_i\frac{\partial \varphi^m}{\partial y}\right) \qquad \text{Eqn. S5}$$

The Nernst-Planck equation, describes concentration- and field-driven transport of solute i in a membrane [30]:

$$J_i = -D_i^m\left(\frac{\partial C_i{}^m}{\partial y} - \frac{Fz_iC_i{}^m}{RT}\frac{\partial \varphi^m}{\partial y}\right) + c_i^m v_i \qquad \text{Eqn. S6}$$

The rightmost term describes convective transport, which is absent in solution-diffusion membranes. Simplification and rearrangement yields:

$$J_i = -\frac{D_i^m C_i{}^m}{RT}\left(\frac{RT}{C_i{}^m}\frac{\partial C_i{}^m}{\partial y} - Fz_i\frac{\partial \varphi^m}{\partial y}\right) \qquad \text{Eqn. S7}$$

Comparison of Eqns. S5 and S7 shows that:

$$L = \frac{D_iC_i{}^m}{RT} \qquad \text{Eqn. S8}$$

Combining Eqn. S2 and S8 yields:

$$J_i = -\frac{D_i^m C_i^m}{RT}\frac{\partial \mu_i^m}{\partial y} \qquad \text{Eqn. S9}$$

Invocation of the chain rule permits integration over the membrane (the difference in y-position between the upstream and downstream sides is the membrane thickness, δ_m):

$$J_i = -\frac{D_i^m}{RT}\left[C_i{}^m\frac{\partial \mu_i{}^m}{\partial C_i{}^m}\right]\frac{\partial C_i{}^m}{\partial y} \qquad \text{Eqn. S10}$$

$$\int_0^{\delta m} J_i dy = -\frac{D_i^m}{RT}\int_0^{\delta m}\left[C_i{}^m\frac{\partial \mu_i{}^m}{\partial C_i{}^m}\right] dC_i{}^m \qquad \text{Eqn. S11}$$

When the system is operating at steady state and no accumulation is occurring, according to the conservation of mass, the flux is independent of position. Additionally, we approximate $\frac{\partial \mu_i{}^m}{\partial C_i{}^m}$ with a finite difference, $\frac{\Delta \mu_i{}^m}{\Delta C_i{}^m}$, and assume a linear profile of C_i^m:

$$J_i \Delta y = -\frac{D_i^m}{RT}\frac{\Delta \mu_i{}^m}{\Delta C_i{}^m}\int_0^{\delta m} C_i{}^m dC_i{}^m \qquad \text{Eqn. S12}$$

$$J_i(y_d - y_u) = -\frac{D_i^m}{RT}\frac{\left(\mu_{i,d}^m - \mu_{i,u}^m\right)}{\left(C_{i,d}^m - C_{i,u}^m\right)}\left(\frac{\left(C_{i,d}^m\right)^2}{2} - \frac{\left(C_{i,u}^m\right)^2}{2}\right) \qquad \text{Eqn. S13}$$

where subscripts u and d refer to the upstream and downstream sides of the membrane, respectively, which are defined with respect to the direction of transport of solute *i.* Solutes always transport from the upstream to the downstream side of the membrane.

Eqn. S13 can be simplified further by factoring:

$$J_i(y_d - y_u) = -\frac{D_i^m}{2RT}\frac{\left(\mu_{i,d}^m - \mu_{i,u}^m\right)}{\left(C_{i,d}^m - C_{i,u}^m\right)}\left(C_{i,d}^m - C_{i,u}^m\right)\left(C_{i,d}^m + C_{i,u}^m\right) \qquad \text{Eqn. S14}$$

$$J_i(y_d - y_u) = -\frac{D_i^m\left(\mu_{i,d}^m - \mu_{i,u}^m\right)}{RT}\frac{\left(C_{i,d}^m + C_{i,u}^m\right)}{2} \qquad \text{Eqn. S15}$$

The difference in y-position between the upstream and downstream sides is the membrane thickness, δ_m. The solution-diffusion model widely recognizes diffusion through the polymer to be the limiting step and that the membrane-liquid interface is at equilibrium such that the electrochemical potential in the external solution and membrane phases at the interface are equal (i.e., $\mu_i^s = \mu_i^m$)[28]. Introducing both simplifications gives:

$$J_i\delta_m = -\frac{D_i^m\left(\mu_{i,d}^s - \mu_{i,u}^s\right)}{RT}\frac{\left(C_{i,d}^m + C_{i,u}^m\right)}{2} \qquad \text{Eqn. S16}$$

The use of concentration in the membrane phase is inconvenient, as we often do not have these value available in experimental conditions. At the solution-membrane interface, we should take into account the concentration ratio due to selective sorption of species i. The sorption coefficient, K_i, describes the equilibrium concentrations at the liquid membrane interface as [28]:

$$K_i = \frac{C_i{}^m}{C_i{}^s} \qquad \text{Eqn. S17}$$

Therefore:

$$J_i\delta_m = -\frac{D_i^m K_i\left(\mu_{i,d}^s - \mu_{i,u}^s\right)}{RT}\frac{\left(C_{i,d}^s + C_{i,u}^s\right)}{2} \qquad \text{Eqn. S18}$$

We can apply the concept of permeability to Eqn. S18. Permeability is an intrinsic material property that is determined by the chemistry and structure of the polymer, as well as by the physicochemical properties of the transporting solute [31–35]. The permeability of dense polymeric membranes is described by the solution-diffusion model [36]. In this model, the membrane is treated

as a homogeneous phase into which the solute sorbs or dissolves on the upstream side. The solute then diffuses across the membrane thickness, and desorbs from the membrane on the downstream side[36] This process is commonly represented by the following equation for permeability:

$$P_i = K_i \times D_i \qquad \text{Eqn. S19}$$

Because 'permeability' is sometimes used colloquially to convey other meanings, in the remainder of this work, we add the superscript U (for "universal") to signify membrane permeability *as defined in the solution-diffusion model and with dimensions of length squared per time*, which is universally applicable to any dense polymer membrane. Hence, $P_i = P_i^U$.

Recognizing that the second term on the right hand side of Eqn. S18 is simply the average of the concentration in the solution on either side of the membrane ($\overline{C_i^s}$) and substituting the solution-diffusion model (Eqn. S19) into Eqn. S18 yields:

$$J_i = \frac{P_i^U\left(\mu_{i,u}^s - \mu_{i,d}^s\right)}{RT\delta_m}\overline{C_i^s} \qquad \text{Eqn. S20}$$

which is identical to Eqn. 1 in the main text. Rearranging Eqn. S20 yields a universal equation for P:

$$P_i^U = \frac{J_i RT}{\overline{C_i^s}}\frac{\delta_m}{\left(\mu_{i,u}^s - \mu_{i,d}^s\right)} \qquad \text{Eqn. S21}$$

The advantage of Eqn. S21 is that permeability (a membrane material property) can be determined from measurable experimental conditions (i.e., solution conditions outside the membrane) and flux. It is important to note, however, that the highest accuracy of $\overline{C_i^s}$ requires determination of the solution concentration at the membrane-solution interface, which is often achieved by an estimate of the concentration polarization. Because the information required to make such estimates is not always reported in literature, we neglect concentration polarization in this work and assume the concentration of the solution at the solution-membrane interface is the same as that of the bulk.

Eqn. S21 is equivalent to the widely accepted definition of permeability, i.e., the driving force- and thickness-normalized flux[37], and is a universal metric because it is agnostic to the type of driving force(s). For example, if a concentration difference is the only contributor to the electrochemical potential gradient, Eqn. S21 simplifies to the familiar expression of permeability used in fuel cell and RO applications.[6,37,38] To facilitate cross-application comparisons, conversions between application-specific metrics and the universal permeability metric of Eqn. S21 are included in Section S4. A universal permeability metric enables direct comparison of membrane performance across applications and industries.

Section S4. Conversions between typical figures of merit and universal permeability

Eqn. S21 provides an avenue for calculation of a universal permeability value directly from experimental conditions and observed flux. However, the methods of measuring flux vary by application (e.g., conductivity and salt permeance both describe flux of ions, but in different units), requiring conversion from the flux-related data reported in the literature to the universal permeability metric of Eqn. S21. Table S4 presents the various flux-based measurements documented in the literature, along with the relevant equation for electrochemical potential difference, and finally the conversion from the application-specific metric to the universal permeability. All assumptions, relevant conditions, and considerations for each calculation in each application are discussed in depth in Section S5.

Table S4. Table showing the conversion from application-specific metrics to the universal permeability (Eqn. S21).

Application-specific metric	Symbol	Typical unit	Eqn. for application-specific metric	Electrochemical potential difference	Conversion to P^u [m².s]
Conductivity,	κ	$mS.cm^{-1}$	$\kappa = \frac{J_i \delta_m z_i \mathcal{F}}{\Delta\varphi}$	$\Delta\mu = z_i \mathcal{F} \Delta\varphi$	$P_i^U = \frac{JRT\delta_m}{C_i^s \mathcal{F} z_i \Delta\varphi} = \frac{\kappa RT}{C_i^s \mathcal{F}^2 {z_i}^2}$
Transport number	t	dimensionless	$t_i = \frac{\lvert z_i \rvert J_i}{\sum_j \lvert z_j \rvert J_j} = \frac{\mathcal{F}\lvert z_i \rvert J_i}{I}$	$\Delta\mu = RT\frac{\Delta C}{\bar{C}} + z_i \mathcal{F} \Delta\varphi$	$P_i^U = t_i \frac{\kappa RT}{C_i^s \mathcal{F}^2 {z_i}^2}$
Water permeance	A	$L.m^{-2}.hr^{-1}.bar^{-1}$	$A = \frac{J_i V_i^m}{\Delta\mathbb{P} - \Delta\pi}$	$\Delta\mu = RT\frac{\Delta C}{\bar{C}} + V_i^m \Delta\mathbb{P} = V_i(\Delta\mathbb{P} - \Delta\pi)$	$P_i^U = \frac{J_i RT\delta_m}{C_i^s V_i(\Delta\mathbb{P} - \Delta\pi)} = \frac{ART\delta_m}{C_i^s V_i^2}$
Salt permeance	B	$L.m^{-2}.hr^{-1}$	$B = \frac{J_i}{\Delta C_i}$	$\Delta\mu = RT\frac{\Delta C}{\bar{C}}$	$P_i^U = B\delta_m$
Rejection	Rej	%	$Rej = \left(1 - \frac{C_d^s}{C_u^s}\right) x100\%$	$\Delta\mu = RT\frac{\Delta C}{\bar{C}} + V_i^m \Delta\mathbb{P}$	$P_i^U = \frac{P_w V_w}{RT}(\Delta\mathbb{P} - \Delta\pi)\left(\frac{1}{Rej/100} - 1\right)$
Diffusive permeability	P_i	$m^2.s^{-1}$	$P_i = \frac{J_i \delta_m}{\Delta C_i}$	$\Delta\mu = RT\frac{\Delta C}{C}$	$P_i^U = \frac{J_i \delta_m}{\Delta C_i} = P_i$
Gas permeability	P_i^G	Barrer [$mol.m^{-1}.s^{-1}.Pa^{-1}$]	$P_i^G = \frac{J_i \delta_m}{\Delta\mathbb{P}_i}$	$\Delta\mu = RT\frac{\Delta C}{\bar{C}} + V_i^m \Delta\mathbb{P} = RT\frac{\Delta\mathbb{P}_i}{\mathbb{P}_i}$	$P_i^U = \frac{J_i RT\delta_m}{\Delta\mathbb{P}_i} = P_i^G RT$
Pervaporation separation factor	$\vartheta_{i/j}$	dimensionless	$\vartheta_{i/j} = \frac{J_i}{J_j}\frac{x_j^f}{x_i^f}$	$\Delta\mu = RT\frac{\Delta C}{\bar{C}} + V_i^m \Delta\mathbb{P}$	$P_i^U = \frac{P_j}{\vartheta_{i/j}}\frac{x_i^f}{x_j^f}$

Section S5. Compilation of membrane transport data

This section describes the methods and criteria we used to collect data from the literature and the steps we took to calculate the universal permeability metric.

Scope of data considered

In reviewing data from the literature, we restricted ourselves to data pertaining to dense, homogeneous polymer membranes traditionally described by the solution-diffusion model. Therefore, we excluded studies of nanocomposites, inorganic membranes, membranes with permanent pores, or that relied on convective flow concepts to explain their data.

We sought a diverse dataset that represented multiple membrane types, driving forces, and separations. In general, we prioritized inclusion of studies of commercial membranes in order to make our data as relevant to practical separation technologies as possible. However, due to the lack of available data for some applications, we included a number of laboratory-synthesized membranes as well in order to ensure good representation of different technologies. We also prioritized separations in which the solutes were similar between applications, to better enable a discussion of similarities and differences between the applications. Furthermore, we included separations that supplemented the discussion of other applications. Our intent in this perspective is not to encompass the entire membrane field, but rather to present a universal framework and demonstrate how this framework allows for discussion between multiple applications.

As elaborated below, many studies had to be excluded because they did not report sufficient detail about their experiments to enable us to convert reported membrane performance into the "universal" permeability metric. If the membrane thickness was not reported in the original study, we sometimes used a value obtained from other sources (e.g., selective layer thickness for RO membranes).

In total, our dataset comprises 243 unique P^U values and 140 unique S^U values drawn from 48 different studies. The distribution of values according to application is shown in Figure S1.

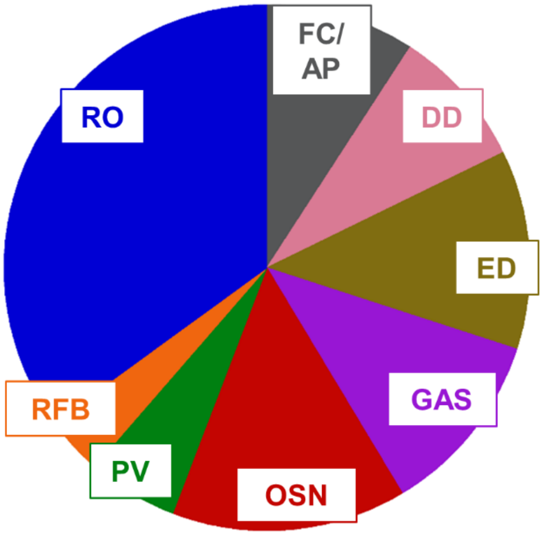

Figure S1. Distribution of data points by application.

Calculation of electrochemical potential difference

For every data point, we calculated the electrochemical potential difference using Eqn. 1 of the main text to express the driving force across the membrane:

$$\Delta\mu_i = RTln\left(\frac{\gamma_{i,d}c_{i,d}}{\gamma_{i,u}c_{i,u}}\right) + \mathcal{F}z_i(\varphi_d - \varphi_u) + V_i(\mathbb{P}_d - \mathbb{P}_u) \qquad \text{Eqn. S17}$$

To do so, we made the following assumptions:

1. We neglected boundary layer effects or concentration polarization that may cause the concentration, pressure, or voltage adjacent to the membrane surface to differ from that in the bulk solution.

2. In general, we assumed unit activity coefficients ($\gamma_i^M = 1$) for dissolved solutes and unit activity ($\gamma_i C_i$ =1) for pure solvents (which corresponds to $\gamma_i^x = 1$ where γ_i^x is expressed on the mole fraction scale, as is common practice for solvents). Non-unit activity coefficients were assigned in selected cases involving very high salt concentrations (for example, some ion exchange membrane salt permeance data were collected with an upstream salt concentration of 4 M NaCl). For solvents, the activity is calculated as the mole fraction of the solvent in the mixture, except for aqueous solutions with a solute concentration >0.5 M, in which case we used the Pitzer model.

3. If the downstream concentration was reported as zero, it was calculated when possible (e.g., from rejection), and when this was not possible it was arbitrarily set to 1 mM.

4. We averaged the upstream and downstream temperatures, implicitly making the assumption that a large temperature difference does not exist across a thin membrane. Upstream and downstream temperatures seldom differed in our dataset.

While these assumptions may limit the accuracy of our conversions in some cases, we believe that they are justifiable and the accuracy adequate for the types of comparisons we present here. Note that the value of $\Delta\mu$ does not affect our estimate of P^U in most cases (see next section). Hence, any inaccuracies introduced by our simplifying assumptions do not necessarily affect the resulting permeability values.

Calculation of universal permeability P^U

We next calculated P^U for each data point. We adopted different approaches depending on the type of data that was available. In selected cases, we were able to compute P^U directly from reported flux and from $\Delta\mu$ using Eq. S3. For all other cases, we converted an application-specific figure of merit to P^U using the conversion formulas shown in Table S4. The following sections elaborate on the specifics of this conversion for each particular application considered, including certain limitations or biases that may arise from the way data are traditionally reported in different fields.

Reverse Osmosis (RO)

Reverse osmosis separation performance is most commonly reported through a combination of water permeance and solute rejection. We compute the downstream (permeate) solute concentration from the reported solute rejection, and then use the upstream and downstream solute concentrations to calculate the difference in water activity (i.e., osmotic pressure, $\Delta\pi$) between the feed and the permeate. The net pressure (applied pressure minus osmotic pressure) is the driving force for water transport, as shown in Table S4. We used the Pitzer model to compute $\Delta\pi$ whenever the upstream solute concentration exceeded 0.5 M, and used the mole fraction of water (equivalent to an osmotic coefficient of unity) otherwise.

We note that the conversion formula listed in Table S4 excludes convective and thermodynamic reference frame corrections.[39,40] Such corrections can be important for precise quantitative treatment of solute permeability in RO membranes, but would not dramatically alter the permeabilities or selectivities of RO membranes in relation to other membrane types we report here.

Organic Solvent Nanofiltration (OSN)

OSN data are generally reported using the same metrics as RO (i.e., solvent permeance and solute rejection), and experimental conditions are typically such that the solutes exist in very dilute concentrations (mM). Hence, we set the solvent activity equal on both sides of the membrane (corresponding to zero osmotic pressure difference) and followed the same procedures used for RO to compute P^U from permeance and solute rejection.

Gas Separation (GAS)

As shown in Table S4, the most common way of reporting solute transport through a membrane in gas separation is gas permeability, which only differs from P^U by a factor of RT. Therefore, no assumptions were necessary to convert the traditionally reported gas permeability to the universal permeability metric.

Gas transport through polymer membranes is usually measured using a constant-volume, variable pressure method;[41] the change in pressure with time is used to calculate the permeability. Because temperature can affect the mobility of the polymer chain segments within the membrane, (and therefore the permeability),[42] we only included data with temperatures in the range of 22-35 °C.

Additionally, we recognize that CO_2 (and some other solutes) plasticizes polymeric materials, and membranes exposed to a higher partial pressure of CO_2 often exhibit a lower selectivity.[43] However, CO_2 plasticization often impacts the selectivity of many common membrane materials on the order of approximately a factor of 3, a small difference relative to the orders-of-magnitude differences in selectivity among applications shown in Fig. 1. Because materials for gas separation are still primarily characterized by pure gas permeabilities and calculated selectivities,[6,43,44] we use pure gas permeabilities for our analysis herein.

Pervaporation (PV)

In alcohol dehydration, which is the most common application of pervaporation, the metrics typically reported are water flux and the separation factor of water over alcohol, defined as:[45–47]

$$\vartheta_{H2O/\text{alcohol}} = \frac{x^p_{H2O}}{x^p_{\text{alcohol}}}\frac{x^f_{alcohol}}{x^f_{H2O}} = \frac{J_{H2O}}{J_{alcohol}}\frac{x^f_{alcohol}}{x^f_{H2O}} \qquad \text{Eqn. S18}$$

where x is the mole or mass fraction and f and p refer to the feed and permeate, respectively. Since the downstream concentration in this process is close to zero for both species, the ratio of fluxes may be equated to the ratio of mole fractions.

In many cases, the feed composition was specified as a weight percent (e.g. 95% ethanol, 5% water) rather than a mole fraction. Such data were converted to mole fractions using the molecular weights of the alcohol and water, and then into molar units using the densities obtained from Table 1 of Chapman et al.[45]

To obtain the downstream concentrations, we started with the separation factor definition:

$$\vartheta_{i/j}\frac{x^f_i}{x^f_j} = \frac{x^p_i}{x^p_j} \qquad \text{Eqn. S19}$$

For a two-component system:

$$x^p_i + x^p_j = 1 \qquad \text{Eqn. S20}$$

Therefore:

$$x_j^p = \frac{1}{\left(1+\vartheta_{i/j}\frac{x_i^f}{x_j^f}\right)}$$ Eqn. S21

Because the downstream phase is vapor, downstream concentrations were converted to mol.L^{-1} based on the molar volume of an ideal gas at the same pressure and temperature as the feed. To convert these data to P^U we employ (see Table S2):

$$P_i^U = \frac{P_j}{\vartheta_{i/j}}\frac{x_i^f}{x_j^f}$$ Eqn. S22

For water / NaCl separation by pervaporation, performance is typically reported in terms of salt rejection, while the upstream salt concentration is given in molar units. Hence, we calculate the downstream concentration from the upstream concentration and the rejection, as in RO. Salt rejection is measured after condensing the permeate vapor, so we convert the downstream salt concentration into a mole fraction using the molarity of pure water (55.5 M). In all cases where PV was used to separate water and NaCl, the salt rejection was extremely high (> 99%) and the downstream phase consisted of (nearly) pure water vapor.

Fuel Cells and Artificial Photosynthesis devices (FC/AP)

Fuels cells and artificial photosynthesis devices are grouped together in this work because membranes for each have essentially the same transport requirements. In both cases, the rejected solute is an uncharged fuel (e.g., methanol) and the permeated solute is a charge carrier (e.g., hydroxide). Due to the relative immaturity of artificial photosynthesis devices compared to fuel cells, most of the data collected from the literature are related to fuel cells. We include the artificial photosynthesis application here to communicate that the discussion around fuel cells can also apply to artificial photosynthesis devices.

In both applications, selectivity is defined as the ratio of the conductivity of the charge-carrying species to the permeability of the uncharged fuel species.[48] These two properties are usually measured independently in *ex situ* measurements, and each is discussed below in detail. In fact, few studies reported both the conductivity and permeability, and hence it was often necessary to use data drawn from different publications to calculate selectivity. When combining data from different publications into one selectivity value, we ensured that the measurements were collected at a similar temperature (maximum difference in temperature is 5 °C) and at a similar degree of hydration (e.g., saturated air, aqueous solution with a low ionic strength). We encourage future materials development publications to include both conductivity and permeability data as both are central to evaluating the performance of the membrane.

Neutral species transport

The transport of neutral species for FC and AP applications is most commonly measured using a diffusion cell. In using diffusion-cell measurements to describe the transport of uncharged solutes, we are neglecting contribution of electro-osmosis to the transport.[49]

Conductivity
The conductivity of ion exchange membranes for fuel cells and artificial photosynthesis is obtained from the Ohmic resistance. Ohmic resistance is often measured via electrochemical impedance spectroscopy, which measures the transport of charge within a polymer membrane in response to an oscillating applied electric field. While there is no single, widely accepted method for this measurement,[50,51] the experimental data presented here were collected according to the following specifications:

- The frequency of alternating current is high enough to prevent the formation of concentration gradients within the material.
- Conductivity is largely independent of the applied potential difference. Many literature references do not report the experimental potential difference. Herein, where the electric potential difference is not reported, we use an arbitrary value of 50 mV, which is in the range commonly used for electrochemical impedance spectroscopy (10 mV to 80 mV).
- Due to the large resistance of dilute aqueous solutions, direct-contact methods of measurement were preferred over difference methods. Direct-contact methods can be made in-plane or through-plane. Because of the aspect ratio difference (electrode contact area vs. distance between electrodes), in-plane measurements were preferred. We recognize that in-plane measurements are made in a direction orthogonal to the direction of transport of the uncharged fuel molecule. We assume that these ion exchange membranes are isotropic, and that the conductivity is the same in any direction. This assumption is reasonable for many commercial ion exchange membranes, though, importantly, not for Nafion.[52,53]
- In direct contact methods of measurement, and at high frequencies, the charged species does not partition between the membrane and the external solution. Therefore, this method only measures transport within the membrane itself. We use conductivity measurements in light of this limitation because conductivity is the most common metric for describing permeability of charged species in response to an electric field. The conductivity measurements presented herein describe diffusion and migration within the polymer and do not include sorption at the polymer interface. Given that counterions commonly have a relatively high sorption coefficient,[54] and that including this factor of sorption will likely impact the selectivity value, we recognize the value of future work in which the sorption coefficient is included in the description of charge transport in response to an electric field.

As such, the concentration term in Eqns. S3 and S21 is the concentration of mobile charge carriers inside the membrane. The external solute concentration can significantly impact conductivity by increasing the number of mobile charge carriers present within the membrane if the external solute concentration is high enough. Because the relationship between the external salt concentration and concentration of mobile charge carriers within the membrane has not yet been studied for many ion exchange

membranes, experimental data collected at low external salt concentrations, in which the salt is largely excluded and the concentration of mobile charge carriers is equal to the fixed charge density of the membrane, were used. While the concentration at which co-ions begin to enter depends on the material, this study considers data from experimental conditions in which the external concentration was less than or equal to 0.3 M.[50]

The fixed charge density (i.e., moles of fixed charge per liter of swollen polymer) is a function of both the polymer and the electrolyte solution, and was chosen over the exchange capacity (IEC, a membrane material property) because fixed charge density better represents the concentration of mobile charge carriers under the above described conditions, and was used for determination of the ion transport properties.[55,56] Where fixed charge density was not present in the literature, IEC was used.

- Some reports describe differences in polymer water uptake between equilibration against liquid water and equilibration against water vapor at 100% relative humidity.[57] While such discrepancies appear to be an experimental artifact[58], conductivity measurements obtained with the membrane submerged in water were preferred due to the prevalence of liquid electrolyte in fuel cell and artificial photosynthesis devices.
- Some of these conductivity measurements were collected in the presence of an uncharged organic solute (e.g., methanol). This detail is important in understanding membrane performance as it may contribute to variations in membrane swelling, but it plays no part in the calculations.

<u>Electrodialysis (ED)</u>

Ion exchange membrane permeabilities to counter- and co-ions were obtained from: 1) conductivity measurements, 2) reported transport numbers and current densities, or 3) individual ion permeabilities obtained from a combination of concentration and electric field-driven measurements.[59]

For ionic conductivity, we considered data obtained both by impedance spectroscopy and direct current (DC) measurements. In all cases, we have only selected data where the background electrolyte resistance was subtracted. Conductivity data was converted to P^U using the formulae in Table S2. While the counter-ion permeability can be obtained from the conductivity, the permeability to the rejected species (co-ions and/or water) are obtained from the concentration difference and experimentally obtained diffusivities or permeabilities. We adopt this approach because, in a charged ion exchange membrane, co-ions permeate as neutral ion pairs or "mobile salt".[60,61]

In cases where both current density, I, and transport number were reported, we were able to obtain the counter and co-ion fluxes by multiplying the respective transport numbers by the current density (see Table S4). We used this information in conjunction with $\Delta\mu_i$ (see above) to calculate P^U. For $\Delta\mu_i$, we used the current density in conjunction with the membrane conductivity to determine the voltage drop, $\Delta\varphi$, across the membrane thickness.

Diffusion Dialysis (DD)

We report any data that were collected in concentration-driven ion transport measurements (in the absence of another driving force) as "diffusion dialysis." In general, these data comprise 1) water vs. mobile salt transport data through ion exchange membranes from Kingsbury et al.[55,62] and 2) Fe^{2+} vs. H^+ transport through anion exchange membranes.[63–66] Laboratory measurements of this type are typically carried out in batch (i.e., non-steady state) mode, meaning that the concentration gradient, and therefore the flux, is changing throughout the duration of the experiment. As such, time-dependent mass balance equations are used to extract the permeability or permeance from the instantaneous flux. Therefore, instead of calculating P^U from $\Delta\mu_i$ and flux (which are not well-defined), we convert the reported permeability or permeance value according to Table S4.

Redox Flow Battery (RFB)

While a variety of membrane chemistries are being explored for redox flow batteries,[67–69] we chose the all-vanadium redox flow battery (VRFB) as a representative system because it is among the most widely studied technologies[68,70] and therefore it has been readily studied in the literature, providing a sufficient data set for our purposes here. Ion transport within VRFBs is complex, given that the four reactive species (V^{2+}, V^{3+}, VO^{2+}, VO_2^+) of which crossover should be minimized and the charge carrier (H^+) are all subject to both concentration gradients and an applied electric field. The situation is further complicated by the changes in the direction of the electric field between charging and discharging, meaning that at times migration occurs in the same direction as diffusion, while at other times migration occurs in the opposite direction.[71] In this work, we adopt a simplified description of transport within VRFBs that considers conductivity for the charge-carrying species and diffusive transport for the reactive species, as is common in membrane development studies for VRFB applications. In material-development publications, vanadium crossover is often measured in a diffusion cell, in the absence of an applied electric field, while proton transport is quantified by conductivity measurements. The selectivity is commonly reported as a ratio of the proton conductivity to the vanadium permeability.[68,72] Due to the requirement of electroneutrality, the vanadium species diffuses through the membrane as a "mobile salt" (e.g., $VOSO_4$) rather than the vanadium ion by itself. For this reason, the separation we include in this study is $H^+/VOSO_4$. Measurement of concentration gradient-driven diffusion also neglects the contribution of electro-osmosis, which can be significant.[70] In the future, expanding on our work to include the impact of electric-field driven transport on reactive species, which has been identified as significant in recent work, would be valuable.[70,71,73]

Section S6. Converting common driving forces to electrochemical potential

Figure 3 was developed by calculating the electrochemical potential difference produced by various driving forces. While recognizing that multiple driving forces can contribute to the electrochemical potential (Eqn. S1), here we discuss each contribution individually. We are only concerned here with the driving force magnitude, not its direction. All calculations were performed for 25°C. For further development of each driving force, we direct the reader to Wesselingh and Krishna's text on mass transfer.[74]

The contribution of electric potential to the electrochemical potential is:

$$\Delta\mu = zF\Delta\varphi \qquad \text{Eqn. S23}$$

Herein, we are considering a monovalent ion (z = 1) that is outside the double layer near the electrode surface. The electric potential applied to the electrodes, therefore, is expected to be greater than this value. An electric potential of 0.5 - 1.0 V per cell pair is typical for ED systems.[75–77]

The contribution of solute concentration to the electrochemical potential is:

$$\Delta\mu = RT(lnC_d^s - lnC_u^s) \qquad \text{Eqn. S24}$$

The example application for this calculation is a direct methanol fuel cell. While the upstream methanol concentration is set to 2 M,[78] determining the downstream concentration is challenging. In DMFCs, the downstream methanol concentration is initially 0 M. However, given the logarithmic nature of the electrochemical potential variation with concentration, a non-zero downstream concentration is required to calculate the electrochemical potential. Therefore, we arbitrarily chose a downstream concentration of 1 mM for this calculation.

The contribution of pressure acting on a compressible fluid to the electrochemical potential is:

$$\Delta\mu = RT\left(ln\mathbb{P}_{i,d}^s - ln\mathbb{P}_{i,u}^s\right) \qquad \text{Eqn. S25}$$

The example application for this calculation is a natural gas separation process. For a single-stage separation, common upstream and downstream pressures are 55.7 bar and 1.7 bar, respectively.[43]

The contribution of pressure acting on an incompressible fluid to the electrochemical potential is:

$$\Delta\mu = V_i\Delta\mathbb{P} \qquad \text{Eqn. S26}$$

The example application for this calculation is water in an RO processes. In this context, V_i is the molar volume of water (18 $cm^3.mol^{-1}$) and a typical transmembrane pressure for brackish water desalination is 55 bar.[6,79]

Also included for each driving force in Figure 3 is a vertical dotted line that represents an order of magnitude decrease in the upstream potential while holding the downstream potential constant. For example, for the case of a concentration driving force, the dotted line represents an electrochemical potential resulting from an upstream concentration of 0.2 M and a downstream concentration of 1 mM.

Section S7. Developing concentration-normalized flux vs. driving force plots

Fig. 4 plots the concentration-normalized flux of a solute as the function of driving force and material properties, offering a graphical representation of Eqn. 4. To validate the use of this graphical representation, we collected water flux data for an RO membrane (Filmtec SW30) under a range of conditions that differed in salt concentration and feed pressure.[6] We plotted the reported flux values against their associated driving forces, calculated from the specified conditions. As shown in Fig. S2, the data exhibit a linear trend. The P_i^U line in Fig. S2 is calculated by multiplying the driving force determined from process conditions by the universal permeability of water in the same membrane measured 20 years later.[80] The slopes of the model line and the P_i^U line are within approximately 25% of one another, which is reasonable considering the long time between measurements and the inherent variability in membrane performance. The relative consistency of the two studies and the linearity of the data suggest that this graphical representation of membrane performance is valid for the purpose of this study.

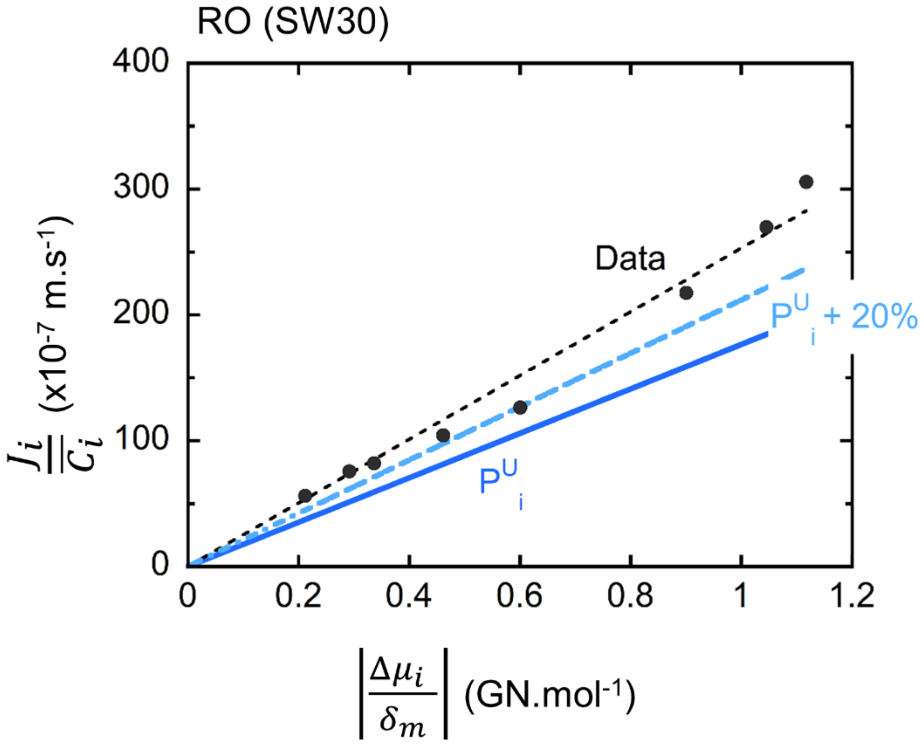

Figure S2. Validation of the graphical representation of Fig. 4 using flux data for a Filmtec SW30 RO membrane.

Case studies

Reverse Osmosis: We assumed a constant salt rejection with varying driving force, except for a slight decrease in rejection when the concentration was significantly greater or the pressure significantly lower than the literature value. Osmotic pressures were determined using the Pitzer model.

Typical industrial conditions:[80]

	**Concentration of NaCl (M)**	**Pressure**
**Upstream**	0.55	55 bar
**Downstream**	0.002	0

Electrodialysis: Osmotic pressure was determined using the Pitzer model.

Typical industrial conditions:

	**Concentration of NaCl (M)**	**Potential**
**Upstream (Diluate)**	0.02	0 V
**Downstream (Concentrate)**	0.2	+/- 0.5 V

Direct methanol fuel cell: The hydroxide flux was calculated directly from the electric potential, assuming a negligible contribution from any concentration gradients. Similarly, electro-osmosis was neglected in methanol transport.

Typical industrial conditions:(41,73)

	**Concentration of MeOH (M)**	**Electric Potential**
**Upstream**	2	0.4
**Downstream**	0.001	0

Table S5. Separation factor and contributing factors, typical process conditions, represented by the black diamonds in Fig. 4. Within each separation, the permeating solute is listed on the left.

**Application**	**Gas**		**Reverse Osmosis**		**Fuel Cell**	
Membrane	*Matrimid*		*SW30HR*		*Selemion AMV*	
Solute	CO_2	CH_4	H_2O	NaCl	OH^-	MeOH
P_i^U ($m^2.s^{-1}$)	7.3×10^{-12}	2.1×10^{-13}	3.5×10^{-11}	2.3×10^{-15}	1.0×10^{-10}	1.4×10^{-11}
S^U (Eqn. 5)	35		15,310		7	
$\frac{\Delta\mu_i}{\delta_m}$ ($GN.mol^{-1}$)	0.02	0.12	0.54	145	0.34	0.17
Γ^U (Eqn. 4)	6		57		15	

**Application**	**Electrodialysis (counter-ion / co-ion)**				**Electrodialysis (counter-ion/water)**			
Membrane	*Neosepta AMX*		*Neosepta CMX*		*Neosepta AMX*		*Neosepta CMX*	
Solute	Cl^-	Na^+	Na^+	Cl^-	Cl^-	H_2O	Na^+	H_2O
P_i^U ($m^2.s^{-1}$)	1.1×10^{-10}	9.4×10^{-13}	6.2×10^{-11}	8.1×10^{-13}	1.1×10^{-10}	1.8×10^{-10}	6.2×10^{-11}	2.1×10^{-10}
S^U (Eqn. 5)	114		76		0.4		0.3	
$\frac{\Delta\mu_i}{\delta_m}$ ($GN.mol^{-1}$)	0.32	0.40	0.25	0.32	0.32	0.00011	0.25	0.00009
Γ^U (Eqn. 4)	90		60		1,730		825	

Section S8. Description of tabulated membrane performance data

We provide the tabulated data used to generate the figures presented in the main text as Supporting Information in the form of comma-separated values (.csv) file. Table S6 provides a description of the data contained in each of the respective columns in the file. Blank values indicate that the data were not found or were not necessary for the calculation, whereas zero values were explicitly entered or calculated.

Table S6. Description of each column in the data file.

Column Name	Units	Description
**Unique ID**	text	A random alphanumeric string used to uniquely identify each entry in the dataset. Unique IDs are used to pair information for rejected and permeating species, which are each listed as separate rows in the data file.
**Permeant ID**	text	The Unique ID of the row describing the permeating species in a separation.
**application**	text	A string representing the application relevant to the separation. Possible categories are AP/FC, GAS, ED, PV, OSN, RO, DD, and RFB. NaCl permeation data through ion exchange membranes is listed as DD.
**separation type**	text	Category to which the separation belongs, according to the charges on the permeating and rejected species, with the rejected species listed first. Possible categories are 'uncharged-charged', 'charged-uncharged', 'charged-charged', and 'uncharged-uncharged.'
**separation**	text	A string representing the separation, with the permeating species listed first.
**separation_latex**	text	A LaTeX-formatted string representing the separation, with the permeating species listed first.
**species**	text	Name or chemical formula of the species whose permeability is described by the data in this row.
**species charge**	dimensionless	Signed charge of the species in this row. For weak acids, we generally assign the charge number based on the dominant species at the listed process conditions.
**species size (ang)**	Å	Radius of the species in this row in angstroms. See Section S2.
**species polarizability (ang**3)**	$Å^3$	Static polarizability of the rejected species in $Å^3$. See Section S2.
**permeant**	text	Name or chemical formula of the permeating species involved in the separation for which the 'species (the species in this row) is the rejected species.
**permeant charge**	dimensionless	Signed charge of the corresponding permeating species.
**permeant size (ang)**	Å	Radius of the permeating species in angstroms. See Section S2.
**permeant polarizability (ang**3)**	$Å^3$	Static polarizability of the permeating species in $Å^3$. See Section S2.

**membrane**	text	Name or description of the membrane used
**thickness (um)**	μm	Membrane thickness in μm, when reported. For reverse osmosis membranes, we assign a thickness of 0.1 μm (100 nm) to represent the active layer thickness, based on literature.[81,82]
**temperature (degK)**	K	Feed temperature in Kelvin. Generally, the feed and permeate temperatures are reported or assumed to be the same. In cases of difference, we report the average temperature, implicitly assuming that there is not a large discontinuity in temperature across a thin membrane.
**molar volume (cm**3/mol)**	$cm^3.mol^{-1}$	Molar volume of the species in $cm^3.mol^{-1}$.
**concentration US (M)**	$mol.L^{-1}$	Upstream solute concentration in $mol.L^{-1}$. For gas separations, the pressure across the membrane is reported as a concentration rather than a pressure. Pressures and mole fractions were converted to molar concentrations using the ideal gas law at the feed temperature.
**concentration DS (M)**	$mol.L^{-1}$	Downstream solute concentration in $mol.L^{-1}$. For gas separations, the pressure across the membrane is reported as a concentration rather than a pressure. Pressures and mole fractions were converted to molar concentrations using the ideal gas law at the feed temperature.
**activity US (-)**	dimensionless	Upstream solute activity on the molar scale (dimensionless). In the majority of cases, we assume ideality (i.e., unit activity coefficients for dissolved solutes; activities equal to one for solvents)
**activity DS (-)**	dimensionless	Downstream solute activity on the molar scale (dimensionless). In the majority of cases, we assume ideality (i.e., unit activity coefficients for dissolved solutes; activities equal to one for solvents)
**pressure US (bar)**	bar	Upstream pressure acting on the solute (bar).
**pressure DS (bar)**	bar	Downstream pressure acting on the solute (bar).
**potential US (V)**	Volts	Electric potential of the upstream feed solution (V). In cases where the permeability calculation is based on a conductivity measurement, we assigned a potential of 50 mV because this value is well within the ohmic regime employed in most measurement techniques.
**potential DS (V)**	Volts	Electric potential of the upstream feed solution (V). In cases where the permeability calculation is based on a conductivity measurement, we assigned a potential of 50 mV because this value is well within the ohmic regime employed in most measurement techniques.
**chempot conc. term (kJ/mol)**	$kJ.mol^{-1}$	Concentration term of the electrochemical potential difference across the membrane, equal to $RT\ln(\gamma_{DS}C_{DS}/\gamma_{US}C_{US})$, in $kJ.mol^{-1}$. See Eqn. 2 in the main text.
**chempot PV term (kJ/mol)**	$kJ.mol^{-1}$	Pressure-volume term of the electrochemical potential difference across the membrane, equal to $V_i(P_{DS} - P_{US})$, in $kJ.mol^{-1}$. See Eqn. 2 in the main text.

**chempot elec. term (kJ/mol)**	$kJ.mol^{-1}$	Electric potential term of the electrochemical potential difference across the membrane, equal to $\mathcal{F}z_i(\varphi_{DS} - \Phi_{US})$, in $kJ.mol^{-1}$. See Eqn. 2 in the main text.
**chempot difference (kJ/mol)**	$kJ.mol^{-1}$	Total difference in electrochemical potential across the membrane, in $kJ.mol^{-1}$. This column is the sum of chempot conc. Term, chempot PV term, and chempot, elec. Term. See Eqn. 2 in the main text.
**chempot gradient (kJ/mol/um)**	$kJ.mol^{-1}$	Electrochemical potential gradient across the membrane, in $kJ.mol^{-1}.\mu m^{-1}$, calculated as chempot difference divided by thickness.
**P_universal (m**2/s)**	$m^2.s^{-1}$	Solute permeability in $m^2.s^{-1}$, calculated according to Eqn. 4 in the main text.
**permeant P_universal (m**2/s)**	$m^2.s^{-1}$	Permeant permeability in $m^2.s^{-1}$, calculated according to Eqn. 4 in the main text.
**selectivity (-)**	dimensionless	Selectivity for the separation defined by the species and the permeating species, calculated according to Eqn. 6 of the main text.
**separation factor (-)**	dimensionless	Separation factor for the separation defined by the species and the permeating species, calculated according to Eqn. 5 of the main text.
**reference**	text	Citation for the original data

Section S9. Histograms of Permeability Data

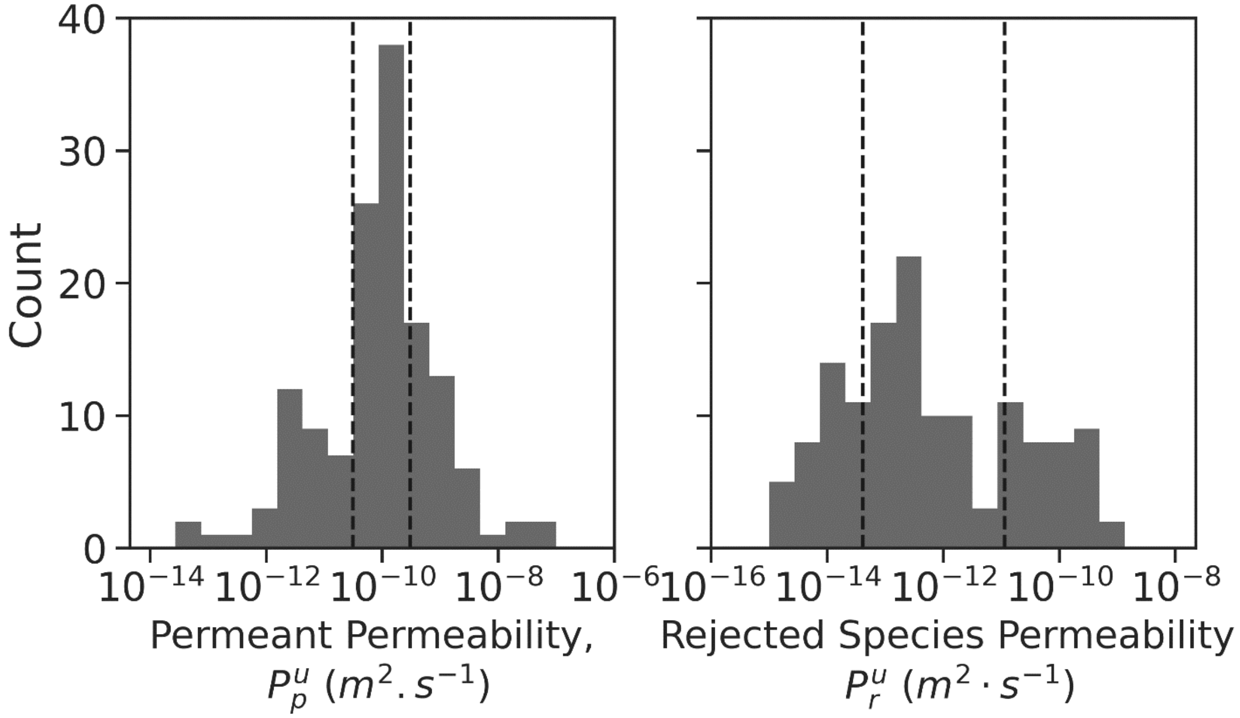

Figure S3. Distribution of universal permeability data for separations shown in Figure 1. Left: permeant permeability P_p^U; Right: rejected species permeability P_r^U. Dashed vertical lines indicate the interquartile range (25th to 75th percentile) of the respective data sets.

References

(1) Epsztein, R.; Shaulsky, E.; Qin, M.; Elimelech, M. Activation Behavior for Ion Permeation in Ion-Exchange Membranes: Role of Ion Dehydration in Selective Transport. *J. Membr. Sci.* **2019**, *580* (January), 316–326. https://doi.org/10.1016/j.memsci.2019.02.009.

(2) Lu, C.; Hu, C.; Ritt, C. L.; Hua, X.; Sun, J.; Xia, H.; Liu, Y.; Li, D.-W.; Ma, B.; Elimelech, M.; Qu, J. In Situ Characterization of Dehydration during Ion Transport in Polymeric Nanochannels. *J. Am. Chem. Soc.* **2021**, *143* (35), 14242–14252. https://doi.org/10.1021/jacs.1c05765.

(3) Hanwell, M. D.; Curtis, D. E.; Lonie, D. C.; Vandermeersch, T.; Zurek, E.; Hutchison, G. R. Avogadro: An Advanced Semantic Chemical Editor, Visualization, and Analysis Platform. *J. Cheminformatics* **2012**, *4* (17). https://doi.org/10.1186/1758-2946-4-17.

(4) Liotard, D. A.; Hawkins, G. D.; Lynch, G. C.; Cramer, C. J.; Truhlar, D. G. Improved Methods for Semiempirical Solvation Models. *J. Comput. Chem.* **1995**, *16* (4), 422–440. https://doi.org/10.1002/jcc.540160405.

(5) Dill, K. A.; Bromberg, S. *Molecular Driving Forces: Statistical Thermodynamics in Biology, Chemisty, Physics, and Nanoscience*; Garland Science, Taylor & Francis Group, LLC: New York, 2011.

(6) Baker, R. W. *Membrane Technology and Applications*, Third Edit.; John Wiley & Sons, Ltd, 2012.

(7) Freeman, B. D. Basis of Permeability/Selectivity Tradeoff Relations in Polymeric Gas Separation Membranes. *Macromolecules* **1999**, *32* (2), 375–380. https://doi.org/10.1021/ma9814548.

(8) Nightingale, R. Phenomenological Theory of Ion Solvation. Effective Radii of Hydrated Ions. *J. Phys. Chem.* **1959**, *63*, 1381–1387.

(9) Liu, Z.; Li, R.; Chen, J.; Wu, X.; Zhang, K.; Mo, J.; Yuan, X.; Jiang, H.; Holze, R.; Wu, Y. Theoretical Investigation into Suitable Pore Sizes of Membranes for Vanadium Redox Flow Batteries. *ChemElectroChem* **2017**, *4* (9), 2184–2189. https://doi.org/10.1002/celc.201700244.

(10) Saitua, H.; Gil, R.; Padilla, A. P. Experimental Investigation on Arsenic Removal with a Nanofiltration Pilot Plant from Naturally Contaminated Groundwater. *Desalination* **2011**, *274* (1–3), 1–6. https://doi.org/10.1016/j.desal.2011.02.044.

(11) Corti, H.; Crovetto, R.; Fernández-Prini, R. Properties of the Borate Ion in Dilute Aqueous Solutions. *J. Chem. Soc. Faraday Trans. 1 Phys. Chem. Condens. Phases* **1980**, *76* (1), 2179–2186. https://doi.org/10.1039/F19807602179.

(12) Marcus, Y. *Ions in Solution and Their Solvation*; 2015. https://doi.org/10.1002/9781118892336.

(13) Tu, K. L.; Fujioka, T.; Khan, S. J.; Poussade, Y.; Roux, A.; Drewes, J. E.; Chivas, A. R.; Nghiem, L. D. Boron as a Surrogate for N -Nitrosodimethylamine Rejection by Reverse Osmosis Membranes in Potable Water Reuse Applications. *Environ. Sci. Technol.* **2013**, *47* (12), 6425–6430. https://doi.org/10.1021/es400732x.

(14) Van Der Bruggen, B.; Schaep, J.; Wilms, D.; Vandecasteele, C. Influence of Molecular Size, Polarity and Charge on the Retention of Organic Molecules by Nanofiltration. *J. Membr. Sci.* **1999**. https://doi.org/10.1016/S0376-7388(98)00326-3.

(15) Chenyakin, Y.; Ullmann, A. D.; Evoy, E.; Renbaum-Wolff, L.; Kamal, S.; Bertram, K. A. Diffusion Coefficients of Organic Molecules in Sucrose-Water Solutions and Comparison

with Stokes-Einstein Predictions. *Atmospheric Chem. Phys.* **2017**, *17* (3), 2423–2435. https://doi.org/10.5194/acp-17-2423-2017.
(16) Xu, Y. C.; Wang, Z. X.; Cheng, X. Q.; Xiao, Y. C.; Shao, L. Positively Charged Nanofiltration Membranes via Economically Mussel-Substance-Simulated Co-Deposition for Textile Wastewater Treatment. *Chem. Eng. J.* **2016**, *303*, 555–564. https://doi.org/10.1016/j.cej.2016.06.024.
(17) Lo Nostro, P.; Ninham, B. W. Hofmeister Phenomena: An Update on Ion Specificity in Biology. *Chem. Rev.* **2012**, *112* (4), 2286–2322. https://doi.org/10.1021/cr200271j.
(18) Parsons, D. F.; Boström, M.; Nostro, P. Lo; Ninham, B. W. Hofmeister Effects: Interplay of Hydration, Nonelectrostatic Potentials, and Ion Size. *Phys. Chem. Chem. Phys.* **2011**, *13* (27), 12352–12367. https://doi.org/10.1039/c1cp20538b.
(19) Rozsa, V. F.; Galli, G. Molecular Polarizabilities in Aqueous Systems from First-Principles. *J. Phys. Chem. B* **2021**. https://doi.org/10.1021/acs.jpcb.0c10732.
(20) Parsons, D. F.; Ninham, B. W. Charge Reversal of Surfaces in Divalent Electrolytes: The Role of Ionic Dispersion Interactions. *Langmuir* **2010**, *26* (9), 6430–6436. https://doi.org/10.1021/la9041265.
(21) Li, M.; Zhuang, B.; Lu, Y.; Wang, Z. G.; An, L. Accurate Determination of Ion Polarizabilities in Aqueous Solutions. *J. Phys. Chem. B* **2017**, *121* (26), 6416–6424. https://doi.org/10.1021/acs.jpcb.7b04111.
(22) Molina, J. J.; Lectez, S.; Tazi, S.; Salanne, M.; Roques, J.; Simoni, E.; Madden, P. A.; Turq, P. Ions in Solutions : Determining Their Polarizabilities from First-Principles. *J. Chem. Phys.* **2011**, *134* (014511), 1–6. https://doi.org/10.1063/1.3518101.
(23) Miller, K. J. Additivity Methods in Molecular Polarizability. *J. Am. Chem. Soc.* **1990**, *112* (23), 8533–8542. https://doi.org/10.1021/ja00179a044.
(24) Wishart, David. FooDB: The Food Database, 2020. https://foodb.ca/compounds/FDB014459.
(25) Djorović, A.; Meyer, M.; Darby, B. L.; Le Ru, E. C. Accurate Modeling of the Polarizability of Dyes for Electromagnetic Calculations. *ACS Omega* **2017**, *2* (5), 1804–1811. https://doi.org/10.1021/acsomega.7b00171.
(26) Calori, I. R.; Pellosi, D. S.; Vanzin, D.; Cesar, G. B.; Pereira, P. C. S.; Politi, M. J.; Hioka, N.; Caetano, W. Distribution of Xanthene Dyes in DPPC Vesicles: Rationally Accounting for Drug Partitioning Using a Membrane Model. *J. Braz. Chem. Soc.* **2016**, *27* (11), 1938–1948. https://doi.org/10.5935/0103-5053.20160079.
(27) Wesselingh, J. A.; Krishna, R. *Mass Transfer in Multicomponent Mixtures*, 1st ed.; Delft University Press, 2000.
(28) Wijmans, J. G.; Baker, R. W. The Solution-Diffusion Model: A Review. *J. Membr. Sci.* **1995**, *107* (1–2), 1–21. https://doi.org/10.1016/0376-7388(95)00102-I.
(29) Bird, R. B.; Stewart, W. E.; Lightfoot, E. N. *Transport Phenomena*, 2nd ed.; John Wiley & Sons, Inc., 2007.
(30) Kamcev, J.; Freeman, B. D. Charged Polymer Membranes for Environmental/Energy Applications. *Annu. Rev. Chem. Biomol. Eng.* **2016**, *7* (1), 111–133. https://doi.org/10.1146/annurev-chembioeng-080615-033533.
(31) Pönitsch, M.; Kirchheim, R. Relation between Prefactor and Activation Energy for the Diffusion of Atoms and Small Molecules in Polymers. *Scr. Mater.* **1996**, *34* (9), 1479–1482. https://doi.org/10.1016/1359-6462(96)00011-5.

(32) Hofmann, D.; Fritz, L.; Ulbrich, J.; Paul, D. Molecular Simulation of Small Molecule Diffusion and Solution in Dense Amorphous Polysiloxanes and Polyimides. *Comput. Theor. Polym. Sci.* **2000**, *10* (5), 419–436. https://doi.org/10.1016/S1089-3156(00)00007-6.
(33) Hofmann, D.; Fritz, L.; Ulbrich, J.; Schepers, C.; Bhning, M. Detailed-Atomistic Molecular Modeling of Small Molecule Diffusion and Solution Processes in Polymeric Membrane Materials. *Macromol. Theory Simul.* **2000**, *9* (6), 293–327. https://doi.org/10.1002/1521-3919(20000701)9:6<293::AID-MATS293>3.0.CO;2-1.
(34) Hofmann, D.; Heuchel, M.; Yampolskii, Y.; Khotimskii, V.; Shantarovich, V. Free Volume Distributions in Ultrahigh and Lower Free Volume Polymers: Comparison between Molecular Modeling and Positron Lifetime Studies. *Macromolecules* **2002**, *35* (6), 2129–2140. https://doi.org/10.1021/ma011360p.
(35) Hölck, O.; Siegert, M. R.; Heuchel, M.; Böhning, M. CO2 Sorption Induced Dilation in Polysulfone: Comparative Analysis of Experimental and Molecular Modeling Results. *Macromolecules* **2006**, *39* (26), 9590–9604. https://doi.org/10.1021/ma061562r.
(36) Wijmans, J. G.; Baker, R. W. The Solution-Diffusion Model: A Review. *J. Membr. Sci.* **1995**, *107*, 1–21. https://doi.org/10.1016/0376-7388(95)00102-I.
(37) Geise, G. M.; Lee, H.-S.; Miller, D. J.; Freeman, B. D.; McGrath, J. E.; Paul, D. R. Water Purification by Membranes: The Role of Polymer Science. *J. Polym. Sci. Part B Polym. Phys.* **2010**, *48*, 1685–1718. https://doi.org/10.1002/polb.
(38) Dischinger, S. M.; Gupta, S.; Carter, B. M.; Miller, D. J. Transport of Neutral and Charged Solutes in Imidazolium- Functionalized Poly (Phenylene Oxide) Membranes for Artificial Photosynthesis. *Ind. Eng. Chem. Res.* **2019**. https://doi.org/10.1021/acs.iecr.9b05628.
(39) Zhang, H.; Geise, G. M. Modeling the Water Permeability and Water/Salt Selectivity Tradeoff in Polymer Membranes. *J. Membr. Sci.* **2016**, *520*, 790–800. https://doi.org/10.1016/j.memsci.2016.08.035.
(40) Geise, G. M.; Paul, D. R.; Freeman, B. D. Fundamental Water and Salt Transport Properties of Polymeric Materials. *Prog. Polym. Sci.* **2014**, *39* (1), 1–42. https://doi.org/10.1016/j.progpolymsci.2013.07.001.
(41) O'Brien, K. C.; Koros, W. J.; Barbari, T. A. A New Technique for the Measurement of Multicomponent Gas Transport through Polymeric Films. *J. Membr. Sci.* **1986**, *29*, 229–238.
(42) Rowe, B. W.; Robeson, L. M.; Freeman, B. D.; Paul, D. R. Influence of Temperature on the Upper Bound: Theoretical Considerations and Comparison with Experimental Results. *J. Membr. Sci.* **2010**, *360* (1–2), 58–69. https://doi.org/10.1016/j.memsci.2010.04.047.
(43) Baker, R. W.; Lokhandwala, K. Natural Gas Processing with Membranes: An Overview. *Ind. Eng. Chem. Res.* **2008**, *47* (7), 2109–2121. https://doi.org/10.1021/ie071083w.
(44) Robeson, L. M.; Liu, Q.; Freeman, B. D.; Paul, D. R. Comparison of Transport Properties of Rubbery and Glassy Polymers and the Relevance to the Upper Bound Relationship. *J. Membr. Sci.* **2015**, *476*, 421–431. https://doi.org/10.1016/j.memsci.2014.11.058.
(45) Chapman, P. D.; Oliveira, T.; Livingston, A. G.; Li, K. Membranes for the Dehydration of Solvents by Pervaporation. *J. Membr. Sci.* **2008**, *318* (1–2), 5–37. https://doi.org/10.1016/j.memsci.2008.02.061.
(46) Shao, P.; Huang, R. Y. M. Polymeric Membrane Pervaporation. *Journal of Membrane Science*. 2007, pp 162–179. https://doi.org/10.1016/j.memsci.2006.10.043.

(47) Smitha, B.; Suhanya, D.; Sridhar, S.; Ramakrishna, M. Separation of Organic-Organic Mixtures by Pervaporation - A Review. *J. Membr. Sci.* **2004**, *241* (1), 1–21. https://doi.org/10.1016/j.memsci.2004.03.042.
(48) Neburchilov, V.; Martin, J.; Wang, H.; Zhang, J. A Review of Polymer Electrolyte Membranes for Direct Methanol Fuel Cells. *J. Power Sources* **2007**, *169* (2), 221–238. https://doi.org/10.1016/j.jpowsour.2007.03.044.
(49) Varcoe, J. R.; Slade, R. C. T. Prospects for Alkaline Anion-Exchange Membranes in Low Temperature Fuel Cells. *Fuel Cells* **2005**, *5* (2), 187–200. https://doi.org/10.1002/fuce.200400045.
(50) Kamcev, J.; Sujanani, R.; Jang, E.-S.; Yan, N.; Moe, N.; Paul, D. R.; Freeman, B. D. Salt Concentration Dependence of Ionic Conductivity in Ion Exchange Membranes. *J. Membr. Sci.* **2017**. https://doi.org/10.1016/j.memsci.2017.10.024.
(51) Díaz, J. C.; Kamcev, J. Ionic Conductivity of Ion-Exchange Membranes: Measurement Techniques and Salt Concentration Dependence. *J. Membr. Sci.* **2021**, *618*, 118718. https://doi.org/10.1016/j.memsci.2020.118718.
(52) Elabd, Y. A.; Walker, C. W.; Beyer, F. L. Triblock Copolymer Ionomer Membranes Part II . Structure Characterization and Its Effects on Transport Properties and Direct Methanol Fuel Cell Performance. *J. Membr. Sci.* **2004**, *231*, 181–188. https://doi.org/10.1016/j.memsci.2003.11.019.
(53) Cooper, K. R. Characterizing Through-Plane and in-Plane Ionic Conductivity of Polymer Electrolyte Membranes. *ECS Trans.* **2011**, *41* (1), 1371–1380.
(54) Kamcev, J.; Paul, D. R.; Freeman, B. D. Ion Activity Coefficients in Ion Exchange Polymers: Applicability of Manning’s Counterion Condensation Theory. *Macromolecules* **2015**, *48* (21), 8011–8024. https://doi.org/10.1021/acs.macromol.5b01654.
(55) Kingsbury, R.; Zhu, S.; Flotron, S.; Coronell, O. Microstructure Determines Water and Salt Permeation in Commercial Ion-Exchange Membranes. *ACS Appl. Mater. Interfaces 10* (46), 39745–39756. https://doi.org/10.1021/acsami.8b14494.
(56) Kamcev, J.; Paul, D. R.; Manning, G. S.; Freeman, B. D. Predicting Salt Permeability Coefficients in Highly Swollen, Highly Charged Ion Exchange Membranes. *ACS Appl. Mater. Interfaces* **2017**, *9* (4), 4044–4056. https://doi.org/10.1021/acsami.6b14902.
(57) Kusoglu, A.; Weber, A. Z. New Insights into Perfluorinated Sulfonic-Acid Ionomers. *Chem. Rev.* **2017**, *117* (3), 987–1104. https://doi.org/10.1021/acs.chemrev.6b00159.
(58) Beers, K. M.; Yakovlev, S.; Jackson, A.; Wang, X.; Hexemer, A.; Downing, K. H.; Balsara, N. P. Absence of Schroeder’s Paradox in a Nanostructured Block Copolymer Electrolyte Membrane. *J. Phys. Chem. B* **2014**, *118* (24), 6785–6791. https://doi.org/10.1021/jp501374r.
(59) Kamcev, Jovan, Paul, Donald R., Manning, Gerald S., Freeman, B. D. Ion Diffusion Coefficients in Ion Exchange Membranes: Significance of Counter-Ion Condensation. *Macromolecules* **2018**, *51* (15), 5519–5529. https://doi.org/10.1021/acs.macromol.8b00645.
(60) Tedesco, M.; Hamelers, H. V. M.; Biesheuvel, P. M. Nernst-Planck Transport Theory for (Reverse) Electrodialysis: I. Effect of Co-Ion Transport through the Membranes. *J. Membr. Sci.* **2016**, *510*, 370–381. https://doi.org/10.1016/j.memsci.2016.03.012.
(61) Tedesco, M.; Hamelers, H. V. M.; Biesheuvel, P. M. Nernst-Planck Transport Theory for (Reverse) Electrodialysis: II. Effect of Water Transport through Ion-Exchange Membranes. *arxiv* **2016**.

(62) Kingsbury, R. S.; Wang, J.; Coronell, O. Comparison of Water and Salt Transport Properties of Ion Exchange, Reverse Osmosis, and Nanofiltration Membranes for Desalination and Energy Applications. *J. Membr. Sci.* **2020**, *604* (117998). https://doi.org/10.1016/j.memsci.2020.117998.
(63) Luo, J.; Wu, C.; Wu, Y.; Xu, T. Diffusion Dialysis of Hydrochloride Acid at Different Temperatures Using PPO-SiO2 Hybrid Anion Exchange Membranes. *J. Membr. Sci.* **2010**, *347* (1–2), 240–249. https://doi.org/10.1016/j.memsci.2009.10.029.
(64) Palatý, Z.; Bendová, H. Permeability of a Fumasep-FAD Membrane for Selected Inorganic Acids. *Chem. Eng. Technol.* **2018**, *41* (2), 385–391. https://doi.org/10.1002/ceat.201700595.
(65) Palatý, Z.; Bendová, H. Separation of HCl + FeCl2 Mixture by Anion-Exchange Membrane. *Sep. Purif. Technol.* **2009**, *66* (1), 45–50. https://doi.org/10.1016/j.seppur.2008.11.026.
(66) Gueccia, R.; Randazzo, S.; Chillura Martino, D.; Cipollina, A.; Micale, G. Experimental Investigation and Modeling of Diffusion Dialysis for HCl Recovery from Waste Pickling Solution. *J. Environ. Manage.* **2019**, *235* (September 2018), 202–212. https://doi.org/10.1016/j.jenvman.2019.01.028.
(67) Weber, A. Z.; Mench, M. M.; Meyers, J. P.; Ross, P. N.; Gostick, J. T.; Liu, Q. Redox Flow Batteries: A Review. *J. Appl. Electrochem.* **2011**, *41* (10), 1137–1164. https://doi.org/10.1007/s10800-011-0348-2.
(68) Maurya, S.; Shin, S. H.; Kim, Y.; Moon, S. H. A Review on Recent Developments of Anion Exchange Membranes for Fuel Cells and Redox Flow Batteries. *RSC Adv.* **2015**, *5* (47), 37206–37230. https://doi.org/10.1039/c5ra04741b.
(69) Wang, W.; Luo, Q.; Li, B.; Wei, X.; Li, L.; Yang, Z. Recent Progress in Redox Flow Battery Research and Development. *Adv. Funct. Mater.* **2013**, *23* (8), 970–986. https://doi.org/10.1002/adfm.201200694.
(70) Darling, R. M.; Weber, A. Z.; Tucker, M. C.; Perry, M. L. The Influence of Electric Field on Crossover in Redox-Flow Batteries. *J. Electrochem. Soc.* **2016**, *163* (1), A5014–A5022. https://doi.org/10.1149/2.0031601jes.
(71) Luo, Q.; Li, L.; Nie, Z.; Wang, W.; Wei, X.; Li, B.; Chen, B.; Yang, Z. In-Situ Investigation of Vanadium Ion Transport in Redox Flow Battery. *J. Power Sources* **2012**, *218*, 15–20. https://doi.org/10.1016/j.jpowsour.2012.06.066.
(72) Shi, Y.; Eze, C.; Xiong, B.; He, W.; Zhang, H.; Lim, T. M.; Ukil, A.; Zhao, J. Recent Development of Membrane for Vanadium Redox Flow Battery Applications: A Review. *Appl. Energy* **2019**, *238* (November 2018), 202–224. https://doi.org/10.1016/j.apenergy.2018.12.087.
(73) Darling, R. M.; Saraidaridis, J. D.; Shovlin, C.; Fortin, M. Transference Numbers of Vanadium Cations in Nafion. *J. Electrochem. Soc.* **2020**, *167* (2), 020529. https://doi.org/10.1149/1945-7111/ab6b0f.
(74) Wesselingh, J. A.; Krishna, R. *Mass Transfer in Multicomponent Mixtures*, 1st ed.; Delft University Press, 2000.
(75) Hyder, A. H. M. G.; Morales, B. A.; Cappelle, M. A.; Percival, S. J.; Small, L. J.; Spoerke, E. D.; Rempe, S. B.; Walker, W. S. Evaluation of Electrodialysis Desalination Performance of Novel Bioinspired and Conventional Ion Exchange Membranes with Sodium Chloride Feed Solutions. *Membranes* **2021**, *11* (217). https://doi.org/10.3390/ membranes11030217.

(76) Walker, W. S.; Kim, Y.; Lawler, D. F. Treatment of Model Inland Brackish Groundwater Reverse Osmosis Concentrate with Electrodialysis — Part II: Sensitivity to Voltage Application and Membranes. *Desalination* **2014**, *345*, 128–135. https://doi.org/10.1016/j.desal.2014.04.026.
(77) Al-Anzi, B. S.; Dominguez, K. P.; Lienhard, J. H.; McCance, A.; Fernandes, J.; Nayar, K. G.; McGovern, R. K. Cost and Energy Requirements of Hybrid RO and ED Brine Concentration Systems for Salt Production. *Desalination* **2019**, *456* (January), 97–120. https://doi.org/10.1016/j.desal.2018.11.018.
(78) Ahmed, M.; Dincer, I. A Review on Methanol Crossover in Direct Methanol Fuel Cells: Challenges and Achievements. *Int. J. Energy Res.* **2011**, *35*, 1213–1228. https://doi.org/10.1002/er.
(79) Ramon, G. Z.; Wong, M. C. Y.; Hoek, E. M. V. Transport through Composite Membrane, Part 1: Is There an Optimal Support Membrane? *J. Membr. Sci.* **2012**, *415–416*, 298–305. https://doi.org/10.1016/j.memsci.2012.05.013.
(80) Redondo, J.; Busch, M.; Witte, J.-P. D. Boron Removal from Seawater Using FILMTECTM High Rejection SWRO Membranes. *Desalination* **2003**, *156* (1), 229–238. https://doi.org/10.1016/S0011-9164(03)00345-X.
(81) Mi, B.; Coronell, O.; Mariñas, B. J.; Watanabe, F.; Cahill, D. G.; Petrov, I. Physico-Chemical Characterization of NF/RO Membrane Active Layers by Rutherford Backscattering Spectrometry. *J. Membr. Sci.* **2006**, *282* (1–2), 71–81. https://doi.org/10.1016/j.memsci.2006.05.015.
(82) Lin, L.; Feng, C.; Lopez, R.; Coronell, O. Identifying Facile and Accurate Methods to Measure the Thickness of the Active Layers of Thin-Film Composite Membranes - A Comparison of Seven Characterization Techniques. *J. Membr. Sci.* **2016**, *498*, 167–179. https://doi.org/10.1016/j.memsci.2015.09.059.
